# Feasibility, Usability, and Acceptability of a Randomized Controlled Trial Evaluating Teleexercise Interventions for Individuals with Spinal Cord Injury: Interim Analysis of the Spinal Cord Injury Program in Exercise (SCIPE) Study

**DOI:** 10.1016/j.arrct.2025.100495

**Published:** 2025-07-17

**Authors:** Hui-Ju Young, Sangeetha Mohanraj, Laurie A. Malone, Lauren A. Fowler, Tapan S. Mehta, James H. Rimmer, Mohanraj Thirumalai

**Affiliations:** a UAB SHP Research Collaborative, School of Health Professions, University of Alabama at Birmingham, Birmingham, AL; b Department of Occupational Therapy, School of Health Professions, University of Alabama at Birmingham, Birmingham, AL; c Department of Nutrition Sciences, School of Health Professions, University of Alabama at Birmingham, Birmingham, AL; d Department of Family and Community Medicine, School of Medicine, University of Alabama at Birmingham, Birmingham, AL; e Division of Preventive Medicine, School of Medicine, University of Alabama at Birmingham, Birmingham, AL

**Keywords:** Exercise training, Movement-to-music, Rehabilitation, Spinal cord injury, Teleexercise

## Abstract

**Objective:**

To investigate the feasibility, usability, and acceptability of the Spinal Cord Injury Program in Exercise intervention.

**Design:**

Three-arm randomized controlled trial.

**Setting:**

Video-based exercise sessions via an online teleexercise platform.

**Participants:**

Thirty-six participants (N=36) with spinal cord injury (52.8% women, 47.2% men), aged 18-65 years, were randomized to movement-to-music (M2M, n=12), standard exercise training (SET, n=12), or attention control (AC, n=12).

**Interventions:**

M2M and SET participants completed 3 weekly exercise sessions for 8 weeks, while AC participants received weekly educational articles.

**Main Outcome Measures:**

Primary outcomes included feasibility (recruitment and retention rates), platform usability (System Usability Scale, Health IT Usability Evaluation, and qualitative interviews), and acceptability (8-item Physical Activity Enjoyment Scale and qualitative interviews). Preliminary outcomes included changes in physical activity (Leisure Time Physical Activity Questionnaire for People with Spinal Cord Injury) and quality of life (Patient-Reported Outcomes Measurement Information System short forms) at 8 weeks postintervention. Analyses included descriptive statistics, effect size estimation, and qualitative interview analysis.

**Results:**

Follow-up retention at weeks 8, 12, and 16 was 66.7%, 41.7%, and 41.7% for M2M; 83.3%, 66.7%, and 66.7% for SET; and 83.3%, 75.0%, and 66.7% for AC, respectively. The mean ± SD of system usability scale score was 69.8±17.1. SET participants rated the intervention “good,” and M2M and AC rated it “fair.” The mean Health IT usability evaluation scale score of 3.61±0.54 indicated moderate satisfaction, with “Impact” highest and “Perceived Usefulness” lowest. Preliminary outcomes suggested small to moderate improvements in physical activity and quality of life for SET, with effect sizes (Hedge’s *g*) ranging from 0.13 to 0.71.

**Conclusions:**

The interim analysis shows high feasibility and moderate usability and acceptability. Preliminary outcomes suggest potential benefits in physical activity and quality of life, particularly for SET. Future research should focus on enhancing platform usability and long-term participant engagement strategies.

Many people with spinal cord injury (SCI) live in rural or geographically isolated regions where accessing fitness facilities requires significant time and financial resources.^1^ This challenge is a common barrier to exercise for people with physical disabilities.^2^^,^^3^ As a result, exercise remains underutilized among people with SCI, despite its well-established benefits for improving aerobic capacity,^4^ increasing muscular strength,^5^^,^^6^ reducing pain and fatigue, and lowering risk of falls.^7^, ^8^, ^9^, ^10^ In 2020, it was reported that 24.2% of adults aged ≥18 met the 2018 Physical Activity Guidelines for Americans, which included both aerobic and muscle-strengthening activities.^11^ In contrast, a study by Latimer et al^12^ found that people with SCI spent <2% of their waking hours engaged in structured exercise or physical activity. This inactivity can lead to physical deconditioning, creating a detrimental cycle of reduced mobility and increased susceptibility to secondary health conditions.^13^ There is an urgent need to implement effective strategies to promote sustainable exercise behavior in the SCI population.^14^^,^^15^

Several exercise training studies have been conducted with people with SCI, but challenges in reaching and engaging this population persist. These challenges include issues with transportation, accessibility, and the cost of gym memberships.^1^^,^^16^, ^17^, ^18^, ^19^ Teleexercise offers a promising avenue for people with SCI to exercise at home,^20^^,^^21^ thus addressing many of these barriers. A 6-month study found higher satisfaction among participants in telerehabilitation intervention compared to standard care.^22^ With growing internet usage^23^ and advancements in telehealth, scaling tailored teleexercise programs is now feasible,^24^^,^^25^ offering significant potential for conducting high-fidelity SCI exercise trials.

The Spinal Cord Injury Program in Exercise (SCIPE) study aimed to investigate two 8-week teleexercise interventions in adults with SCI: movement-to-music (M2M) and standard exercise training (SET). This report presents the first interim analysis of the SCIPE study, focusing on feasibility, platform usability, and acceptability findings.

## Methods

### Study design

This study was a 3-arm randomized controlled trial with a sequential design and 2 interim analyses. After providing informed consent and completing baseline assessments, eligible and enrolled participants were randomized into 1 of 3 study arms: M2M, SET, and attention control (AC). Data analysts were unaware of study arm assignments. Further details on the study design can be found in the published protocol.^26^

### Participants

Participants were recruited through social media platforms (eg, Instagram, Facebook) and word of mouth. Eligible criteria included: (1) diagnosis of incomplete or complete (C5 and below) SCI; (2) aged 18-65 years; (3) physical activity readiness per the Physical Activity Readiness Questionnaire for Everyone^27^; (4) medical clearance if required by physical activity readiness questionnaire for everyone; and (5) can converse in and read English. Exclusion criteria included: (1) no broadband internet access; (2) significantly low vision that makes it difficult to see a computer screen to follow exercise; and (3) currently pregnant. The study was approved by the university’s Institutional Review Board and registered at ClinicalTrials.gov.

### Interventions

After randomization, participants received an email invitation to the SCIPE platform and were instructed to complete a series of setup steps before beginning the intervention. The steps included (1) creating a password, (2) completing a profile, (3) answering physical function and joint pain questions, and (4) setting an exercise schedule with calendar reminders. For M2M and SET participants, intervention exercises were tailored based on their responses to the physical function and joint pain questions.

The overall structure of the M2M and SET interventions is outlined in table 1. The M2M intervention incorporated choreographed movement sequences set to music, targeting specific fitness components at varying movement tempos. The SET intervention used standard, repetitive exercises. Both interventions were delivered through prerecorded videos 3 times per week for 8 weeks. Sessions progressed from 5 minutes in week 1 to 40 minutes by weeks 7 and 8. Before starting, participants received wrist weights and watched an introductory video on basic movement, posture, equipment use, and safety. Each session began with seated range of motion exercises, followed by muscular strengthening using wrist weights, cardiorespiratory endurance, and balance exercises, performed seated or standing with or without chair support. The session ended with a cool down focusing on breathing. Participants who were able to use both arms and legs to exercise without knee pain received videos demonstrating cardiorespiratory endurance and balance exercises in a standing position. Those with knee pain or those who were able to use both arms to exercise with or without trunk control received videos demonstrating exercises in a seated position. At the beginning of each session, participants with shoulder or back pain watched a safety clip on proper exercise posture. All participants received weekly educational articles through the SCIPE platform, covering topics such as exercise benefits, injury prevention, and goal setting. For a more detailed description of the exercises, please refer to Appendix A and the published study protocol.^26^^,^^27^

**Table 1:** General intervention structure.

Session Component	Session Component Exercise Time							
Wk	1	2	3	4	5	6	7	8
Upper body range of motion	5*	5	5	5	5	5	5	5
Lower body range of motion		5*	5	5	5	5	5	5
Muscular strength			5*	5	5*	5	5*	5
Aerobic				5	5	15*	15	15*
Functional strength			5*	5	5*	5	5*	5
Cool down/breathing				5*	5	5	5	5
Total	5	10	20	30	30	40	40	40

NOTE. Durations (in min) reflect exercise content only and do not include instruction/explanation time.

⁎ Components with newly uploaded videos.

### Outcome measures

The interim analysis focused on the feasibility, platform usability, and acceptability of the SCIPE interventions. Feasibility metrics included recruitment, enrollment, and retention rates across the 8-week intervention and follow-ups at weeks 8 (immediately after intervention completion), 12 (4 wk after intervention), and 16 (8 wk after intervention). Perceived usability was assessed using 2 validated instruments: the System Usability Scale (SUS)^28^ and the Health Information Technology Usability Evaluation Scale (Health-ITUES)^29^^,^^30^ at week 8 postintervention. The SUS is a 10-item questionnaire scored from 0-100, categorized as follows: 100-84.1 (best imaginable), 84.0-80.8 (excellent), 80.7-71.1 (good), 71.0-51.7 (fair), 51.6-25.1 (poor), and ≤25 (worst imaginable). The Health-ITUES consists of 20 items across 4 subscales: *Effect, Perceived Usefulness, Perceived Ease of Use*, and *User Control*. Items are rated on a 5-point Likert scale (1=strongly disagree, 5=strongly agree), with higher scores indicating greater satisfaction and usability. Acceptability was evaluated using the validated 8-item Physical Activity Enjoyment Scale (PACES-8).^31^ The PACES-8 was administered to SET and M2M participants at week 8. Each PACES-8 item is rated on a 7-point Likert scale from 7 (strongly agree) to 1 (strongly disagree), with higher total scores indicating greater enjoyment. Additionally, all participants were invited to participate in semistructured exit interviews after completion of the 8-week intervention, which used open-ended questions to capture participants’ perspectives on platform usability and intervention acceptability. Interviews were audio-recorded and transcribed by a professional transcription service.

### Preliminary study outcomes

Physical activity was evaluated at baseline and week 8 using the Leisure Time Physical Activity Questionnaire for People with Spinal Cord Injury (LTPAQ-SCI),^32^ which captures the frequency (d) and duration (min) of 4 activity categories: (1) mild, (2) moderate, (3) vigorous, and (4) total activities over the past 7 days. Additionally, physical health, health-related quality of life, and social participation were evaluated using Patient-Reported Outcomes Measurement Information System (PROMIS) scales (table 2).^33^, ^34^, ^35^, ^36^, ^37^ Raw PROMIS scores were converted to t-scores (population mean=50, SD=10) using standard scoring guidelines (https://www.healthmeasures.net/).

**Table 2:** Physical health outcomes assessed using PROMIS.

Outcome	Survey Tool
Pain interference^33^	PROMIS Adult Short Form v1.1 - Pain Interference 8a
Pain intensity^34^	PROMIS Adult Short Form v2.0 - Pain Intensity 3a
Sleep disturbance^35^	PROMIS Adult Short Form v1.0 - Sleep Disturbance 8a
Fatigue^36^	PROMIS Adult Short Form v1.0 - Fatigue 8a
Health-related quality of life^37^	PROMIS Scale v1.0 - Global Health
Social participation^37^	PROMIS Short Form v2.0 - Ability to Participate in Social Roles and Activities Adult Short Form 8a

### Statistical analysis

Descriptive statistics for participant demographics, feasibility, usability, acceptability metrics, and preliminary study outcomes were computed as mean ± SD or median (minimum, maximum) for continuous variables and percentage (n) for categorical variables. A sample size of 36 was determined based on recommendations for feasibility studies assessing feasibility, usability, and acceptability.^38^

Preliminary data (LTPAQ-SCI and PROMIS) collected at baseline and week 8 were analyzed, with week 8 as the primary endpoint. Prior to analyses, data were examined for errors, missingness, and normality. Three participants (n=1 in SET, n=2 in AC) were identified as outliers for all LTPAQ-SCI categories (studentized residual >3.0 and high leverage on predictors of interest). These data were presumed to result from measurement errors and were excluded from all LTPAQ-SCI analyses.

Given the exploratory nature of this interim analysis and the small sample size, we focused on effect sizes for the preliminary study outcomes. Linear mixed models were used to assess within-and between-group effects using an intent-to-treat approach and included fixed effects for time (wk 8 vs baseline) and intervention arm, a random effect for participant, and a time × intervention arm interaction. Estimates were derived from least-squares mean differences, with bootstrap confidence intervals (CIs) calculated at the 95%, 85%, and 75% levels. All LTPAQ-SCI measures were square root-transformed for analysis to meet normality assumptions.

Effect sizes were calculated using Hedge’s *g*, which is based on the pooled SD and corrected for small samples. Interpretations of *g* included: *g*<0.2=very small; 0.2≤*g*<0.5=small; 0.5≤*g*<0.8=medium; *g*≥0.8=large.^39^ Rank-biserial correlation index (*r*_rb_) was also used with the following interpretations: 0.05≤*r*_rb_<0.1=very small; 0.1≤*r*_rb_<0.2=small; 0.2≤*r*_rb_<0.3=medium; 0.3≤*r*_rb_<0.4=large; *r*_rb_≥0.4=very large.^40^

Analyses were performed using R version 4.3.3 (R Core Team, 2024)^41^^,a^ and the following packages: tidyverse v2.0.0,^42^ DescTools v0.99.54,^43^ gtsummary v1.72,^44^ lme4 v1.1-35.3,^45^ lmerTest v3.1-3,^46^ emmeans v1.11.0,^47^ effectsize v0.8.7,^48^ broom.mixed v0.2.9.6,^49^ ggplot2 v3.5.2,^50^ and ggpubr v0.6.0.^51^

## Results

Between February 2021 and March 2022, 65 individuals were screened for eligibility. Of these, 29 individuals were excluded for the following reasons: residing outside the United States (n=6), not having a SCI (n=4), injury level above C5 (n=1), being outside the eligible age range (n=1), unable to be reached after expressing interest (n=10), lost interest before screening (n=3), or became lost to contact after eligibility screening (n=4). Ultimately, 36 participants were randomized into 1 of the 3 groups: SET (n=12), M2M (n=12), or AC (n=12). Baseline demographic and health characteristics are summarized in Table 3. Common coexisting health conditions included neuropathy (25.0%), arthritis (11.1%), and incontinence (30.6%).

**Table 3:** Baseline demographic and health-related characteristics of study participants.

Characteristics	Overall (n=36)	AC (n=12)	M2M (n=12)	SET (n=12)
Age, y	45.4±11.7	46.4±12.0	42.0±11.6	47.7±11.8
Sex, % (n)				
Female	52.8 (19)	66.7 (8)	50.0 (6)	41.7 (5)
Male	47.2 (17)	33.3 (4)	50.0 (6)	58.3 (7)
Race, % (n)				
White	72.2 (26)	50.0 (6)	83.3 (10)	83.3 (10)
Black or African American	13.9 (5)	16.7 (2)	8.3 (1)	16.7 (2)
Other*	13.9 (5)	33.3 (4)	8.3 (1)	0.0 (0)
Injury level, % (n)				
Cervical (C3, C5-C7)	25.0 (9)	33.3 (4)	25.0 (3)	16.7 (2)
Thoracic (T2, T5-T12)	55.6 (20)	58.3 (7)	58.3 (7)	50.0 (6)
Lumbar (L1, L3)	8.3 (3)	8.3 (1)	8.3 (1)	8.3 (1)
Do not know/no response	11.1 (4)	0.0 (0)	8.3 (1)	25.0 (3)
Injury type, % (n)				
Paraplegia	69.4 (25)	75.0 (9)	75.0 (9)	58.3 (7)
Quadriplegia	16.7 (6)	16.7 (2)	16.7 (2)	16.7 (2)
No response	13.9 (5)	8.3 (1)	8.3 (1)	25.0 (3)
Injury completeness, % (n)				
Complete	44.4 (16)	41.7 (5)	41.7 (5)	50.0 (6)
Incomplete	47.2 (17)	50.0 (6)	50.0 (6)	41.7 (5)
No response	8.3 (3)	8.3 (1)	8.3 (1)	8.3 (1)
Duration since onset, y	15.8±13.4	17.0±13.7	10.8±8.94	18.9±16.1

⁎ Other races: American Indian/Alaskan Native (n=2 in AC); Asian (n=1 in M2M); unspecified (n=2 in AC).

**Fig 1:**
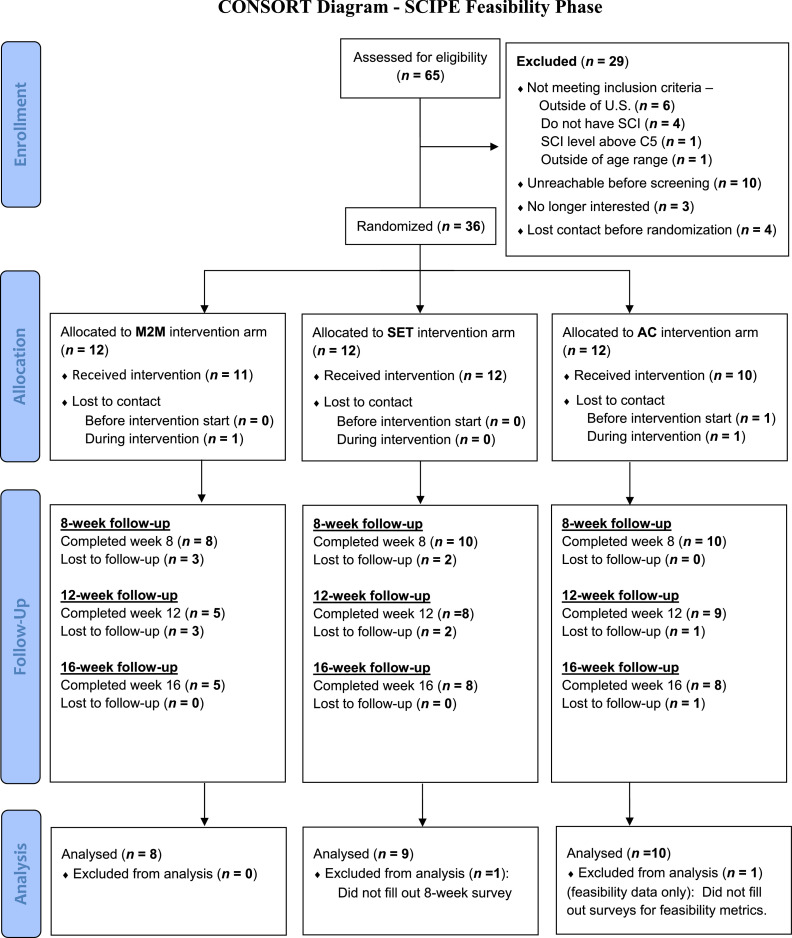
CONSORT diagram reflecting participant flow throughout the study. Partial follow-up indicates participants did not complete entire set of postintervention assessments.

After the intervention, 77.8% completed the postintervention (8-wk) assessment, 61.1% the 12-week follow-up, and 58.3% the 16-week follow-up. Of the 18 participants who completed the 8-week follow-up (n=8 in M2M and n=1 in SET), 8 agreed to participate in the exit interviews (n=5 in SET, n=3 in M2M). Overall, 15 participants were lost to follow-up by week 16 (n=4 in SET [33.3%], n=7 in M2M [58.3%], n=4 in AC [33.3%]). Figure 1 illustrates the participant flow throughout the study.

**Fig 2:**
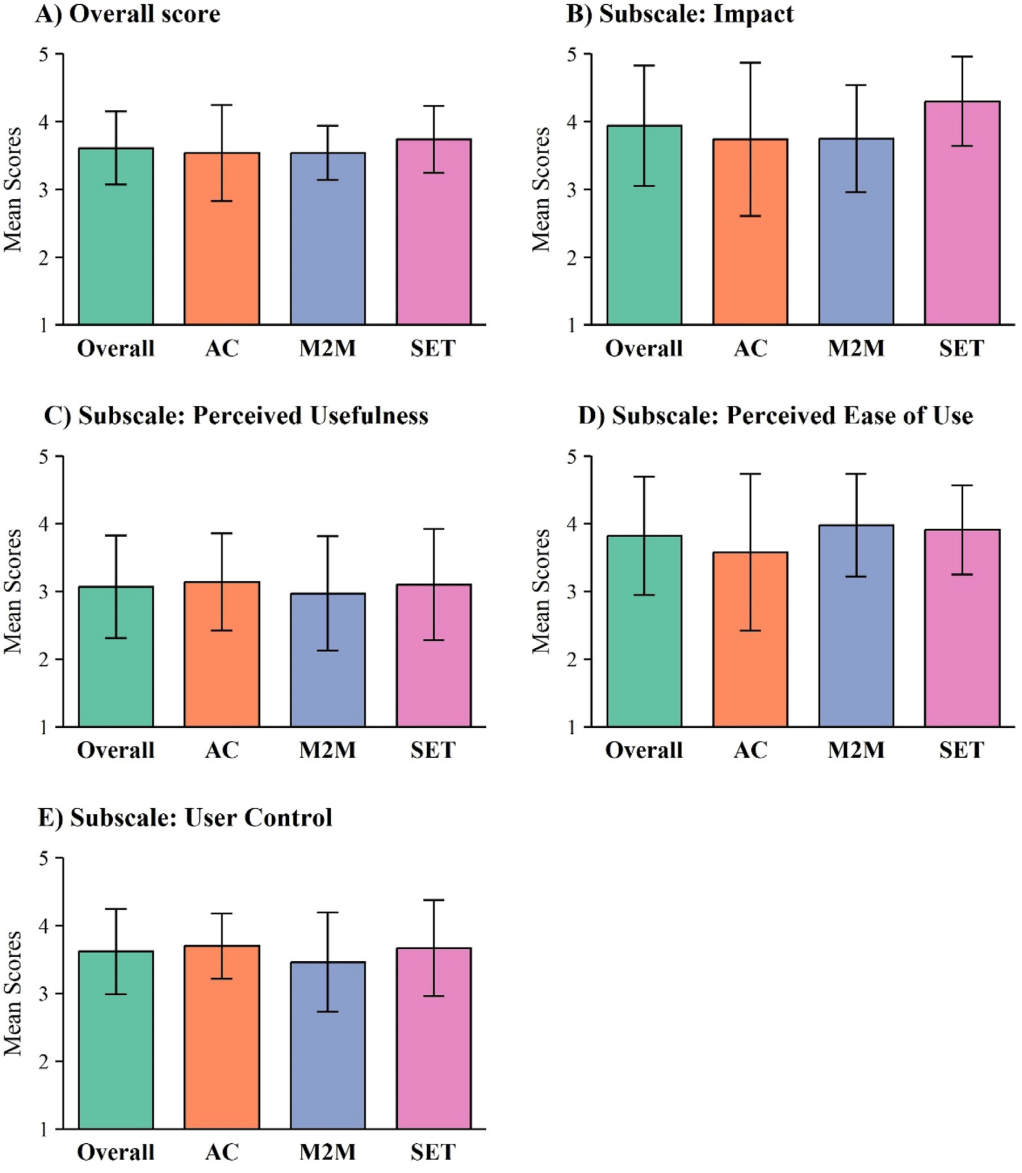
Health-ITUES scores at 8 weeks postintervention across study arms for (A) Overall Score; (B) Effect; (C) Perceived Usefulness; (D) Perceived Ease of Use; and (E) User Control. Data are reported as mean ± SD. Sample sizes were as follows: Overall (n=26), AC (n=9), M2M (n=8), and SET (n=9).

Descriptive results for the usability metrics at 8-week postintervention are presented in table 4. Of the 28 participants who remained in the study at week 8, 26 (92.9%) completed both the SUS and Health-ITUES. The mean SUS score was 69.8±17.1, with scores ranging from 40 (poor usability) to 97.5 (best imaginable usability). SET participants rated the platform usability as “good,” while AC and M2M participants rated it as “fair.” The mean overall Health-ITUES score was 3.61±0.54. As shown in figure 2, among the 26 participants completed the Health-ITUES, the *Effect* subscale was rated the highest (3.94±0.89), followed by *Perceived Ease of Use* (3.82±0.87), *User Control* (3.62±0.63), and *Perceived Usefulness* (3.07±0.76) subscales. SET participants rated *Effect* higher (4.30±0.66) than M2M (3.75±0.79) and AC (3.74±1.13) participants.

**Table 4:** Descriptive statistics for usability metrics at post 8-week intervention.

Statistic	Overall (n=26)	AC (n=9)	M2M (n=8)	SET (n=9)
System usability scale*	69.8±17.1	67.8±16.2	66.6±20.4	74.7±15.7
Health-ITUES†				
Effect	3.94±0.89	3.74±1.13	3.75±0.79	4.30±0.66
Perceived usefulness	3.07±0.76	3.14±0.713	2.97±0.844	3.10±0.823
Perceived ease of use	3.82±0.87	3.58±1.16	3.98±0.759	3.91±0.657
User control	3.62±0.63	3.70±0.484	3.46±0.733	3.67±0.707
Overall score	3.61±0.54	3.54±0.710	3.54±0.402	3.74±0.492

NOTE. Data reported as mean ± SD.

⁎ Interpretation of System Usability Scale scores: 100-84.1 = Best imaginable usability; 84.0-80.8 = Excellent usability; 80.7-71.1 = Good usability; 71.0-51.7 = Fair usability; 51.6-25.1 = Poor usability; ≤25 = Worst imaginable usability.

† Health-ITUES interpretation: Each subscale was rated on a 5-point Likert scale ranging from 5 (strongly agree) to 1 (strongly disagree), with higher scores reflecting greater participant satisfaction and usability. Overall score was calculated as the average of the 4 subscales.

Exit interview responses supported these findings, with most participants describing the SCIPE platform as intuitive and user-friendly (table 5). They highlighted ease of navigation, progress tracking, clean layout, and visually engaging design. Features such as commenting on videos and articles fostered a sense of community. One participant appreciated the ability to resume videos, while another found the social networking feature less useful. Responses also indicated that participants *felt secure using the platform*, citing the absence of intrusive pop-ups, its affiliation with a reputable educational institution, and staff accessibility. They appreciated that the platform did not request personal information, which further reinforced their trust.

**Table 5:** Participant feedback on usability and acceptability of the SCIPE interventions from semistructured exit interviews.

Themes	Quotes
Appreciation for program features	*“It was like I said started slow but then as I wanted it to increase in intensity of the workouts and things it really did. And it was nice to know okay each week is going to be something new but still covering what we had done before. So that was really nice. And then I have an Apple Watch so I was recording. And each week it would increase and everything and that’s exactly what I wanted.” (3682)*
Program expectations and acceptability	*“I think the program itself was great. I was maybe pleasantly surprised, again at the quality of it in terms of the exercise videos and the articles specifically. It probably exceeded expectations.” (3573)* *“I definitely think it met my expectations. Like I said, I had a lot of fun doing it. Going into it I expected exactly what I got out of it. I expected to have a good solid 8 weeks of workouts. That is exactly what I got out of it.” (3655)* *“… my understanding of the study was that it was sort of a practice in exercise with music. And quite honestly, I thought it was boring. The music was boring. The different moves were sort of boring. It was a little bit more dance like than what I like, especially, I don't know, in a wheelchair, I was not expecting to have it be like dance music. You know, I was expecting kind of like upbeat, like really kind of rigorous exercise.” (3666).*
Technology usability and user experience	*“I definitely liked was how easy it was to use, how easy it was to keep track of what you already did, and what you still had to do for a given week.” (3655)* *“I love the app you guys have. …I even liked too if I had to stop for whatever reason and I went back to it, somehow it remembered where I left off….I enjoyed reading the comments from other participants to see how they were going along with the same type of activities that I was doing. So that was probably the uniqueness of it. I did feel connected to the community of participants that were in the SCIPE program at the same time.” (3705)*
Security of intervention platform	*“I would have definitely felt less secure not knowing you guys were connected to something like a university.” (3655)* *“I felt really comfortable and I would contact the administrator for different things... I was surprised that the message didn't come back through the website, it went through email. But once I knew that, that was fine.” (3682)*

For intervention acceptability, the mean PACES-8 score was higher in SET (40.7±10.3) than in M2M (36.6±13.7), with a small effect size (change in least-squares mean = 4.04; 95% CI, −8.42 to 16.5; *g*=−0.32).

### Preliminary effects of the study outcomes

**Fig 3:**
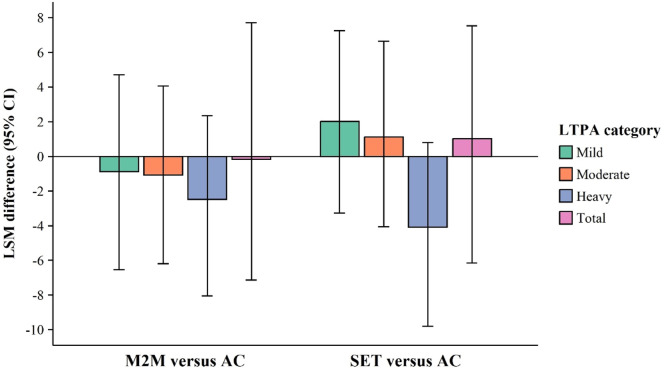
Preliminary estimates for between-group differences of leisure time physical activity in M2M vs AC and SET vs AC. LSM, least-squares means; LTPA, leisure time physical activity.

Estimates for LTPAQ-SCI are presented in table 6, with between-group contrast estimates shown in figure 3. These reflect the differences in changes from baseline to week 8 for M2M vs AC and SET vs AC, along with their corresponding 95% CIs. M2M participants showed consistently minimal changes across all 4 categories, indicating small negative intervention effects (mild: −0.87; 95% CI, −6.54 to 4.70; *g*=−0.12; moderate: −1.07; 95% CI, −6.19 to 4.06; *g*=−0.16; vigorous: −2.48; 95% CI, −8.05 to 2.34; *g*=−0.37; total: −0.174; 95% CI; −7.13 to 7.72; *g*=−0.02). SET participants showed greater increases in mild (2.01; 95% CI, −3.27 to 7.25; *g*=0.28), moderate (1.12; 95% CI, −4.06 to 6.64; *g*=0.17), and total (1.02; 95% CI, −6.15 to 7.53; *g*=0.13) physical activity than AC participants, although still within the small to very small effect range. In contrast, SET participants reported a reduction in vigorous activity, suggesting a moderate negative intervention effect (*g*=−0.62).

**Table 6:** Between-group estimates for LTPAQ-SCI.

		Confidence Intervals for Estimate			Effect Size (Hedge’s *g*)	
Outcome	LSM Difference	95% CI	85% CI	75% CI	Hedge’s *g*	Rank-Biserial Correlation (*r*_rb_)
**Mild LTPA, min/wk**						
Between-group difference for change (time)						
M2M vs AC (ref)	–0.87	(–6.54 to 4.70)	(–4.74 to 3.04)	(–4.08 to 2.25)	–0.12	–0.06
SET vs AC (ref)	2.01	(–3.27 to 7.25)	(–1.99 to 6.33)	(–1.05 to 5.07)	0.28	0.14
Marginal means for change (time)						
SET	3.39	(–0.78 to 7.56)				
M2M	0.51	(–3.71 to 4.74)				
AC	1.38	(–2.52 to 5.28)				
**Moderate LTPA, min/wk**						
Between-group difference for change (time)						
M2M vs AC (ref)	–1.07	(–6.19 to 4.06)	(–4.77 to 2.30)	(–4.57 to 1.74)	–0.16	–0.08
SET vs AC (ref)	1.12	(–4.06 to 6.64)	(–2.85 to 4.76)	(–1.79 to 4.14)	0.17	0.08
Marginal means for change (time)						
SET	2.89	(–1.06 to 6.85)				
M2M	0.71	(–3.22 to 4.63)				
AC	1.78	(–1.85 to 5.40)				
**Heavy LTPA, min/wk**						
Between-group difference for change (Time)						
M2M vs AC (ref)	–2.48	(–8.05 to 2.34)	(–5.92 to 1.33)	(–5.47 to 0.56)	–0.37	–0.18
SET vs AC (ref)	–4.09	(–9.79 to 0.80)	(–7.52 to –0.49)	(–6.99 to –1.12)	–0.62	–0.30
Marginal means for change (Time)						
SET	–0.95	(–4.57 to 2.68)				
M2M	0.67	(–3.11 to 4.44)				
AC	3.14	(–0.41 to 6.70)				
**Total LTPA, min/wk**						
Between-group difference for change (time)						
M2M vs AC (ref)	–0.17	(–7.13 to 7.72)	(–4.58 to 4.71)	(–4.73 to 3.96)	–0.02	–0.01
SET vs AC (ref)	1.02	(–6.15 to 7.53)	(–3.98 to 5.78)	(–2.91 to 4.81)	0.13	0.06
Marginal means for change (time)						
SET	3.74	(–1.50 to 8.98)				
M2M	2.55	(–2.64 to 7.74)				
AC	2.72	(–1.88 to 7.32)				

NOTE. CIs for between-group estimates were calculated using the bootstrap method. Values were square root-transformed for analysis. Estimates reflect the square root-transformed values.

Abbreviations: LSM, least-squares means; LTPA, leisure time physical activity; ref, reference.

From baseline to week 8, SET participants reported the largest increases in mild, moderate, and total physical activity, while M2M participants showed the smallest changes. For vigorous physical activity, AC participants had the greatest increase, followed by M2M, whereas SET reported a decrease.

PROMIS outcome estimates are presented in table 7. SET participants showed a greater reduction in fatigue (−6.71; 85% CI, −12.10 to −1.02) and a larger increase in global physical health (3.22 at 75% CI, 0.41-6.44), each associated with medium effect sizes (*g*=0.71 and *g*=0.51, respectively). Other PROMIS measures in SET reflected small to very small effects (*g* range=0.0002-0.11). When comparing M2M with AC, the between-group differences observed were negligible in magnitude. Pain intensity showed a negligible effect (*g*=0.0004), while other PROMIS outcomes showed small effects (*g* range: 0.27-0.39).

**Table 7:** Between and within-group estimates for PROMIS measures.

		Effect Size (Hedge’s *g*)			
Outcome	LSM Difference	95% CI	85% CI	75% CI	Hedge’s *g*
**Pain interference, T score**					
Between-group difference for change (time)					
M2M vs AC (ref)	2.29	(–2.65 to 6.85)	(–1.39 to 5.44)	(–0.33 to 5.36)	0.39
SET vs AC (ref)	0.62	(–3.99 to 5.19)	(–2.58 to 3.84)	(–1.89 to 3.34)	0.11
Marginal means for change (time)					
SET	–1.57	(–5.00 to 1.85)			
M2M	0.10	(–3.53 to 3.72)			
AC	–2.19	(–5.45 to 1.07)			
**Pain intensity, T score**					
Between-group difference for change (time)					
M2M vs AC (ref)	–0.034	(–6.49 to 6.73)	(–5.42 to 4.93)	(–4.48 to 4.06)	0.000392
SET vs AC (ref)	–0.104	(–7.66 to 6.48)	(–5.14 to 4.60)	(–3.98 to 3.85)	0.01
Marginal means for change (time)					
SET	–1.31	(–6.32 to 3.70)			
M2M	–1.24	(–6.53 to 4.05)			
AC	–1.21	(–5.98 to 3.57)			
**Sleep disturbance, T score**					
Between-group difference for change (time)					
M2M vs AC (ref)	1.52	(–1.64 to 5.27)	(–0.77 to 3.91)	(–0.22 to 3.65)	0.32
SET vs AC (ref)	0.39	(–3.05 to 3.28)	(–2.29 to 2.60)	(–1.45 to 2.36)	0.09
Marginal means for change (time)					
SET	–0.94	(–3.46 to 1.59)			
M2M	0.19	(–2.43 to 2.82)			
Control	–1.33	(–3.77 to 1.11)			
**Fatigue, T score**					
Between-group difference for change (Time)					
M2M vs AC (ref)	–3.23	(–10.45 to 4.49)	(–8.92 to 2.27)	(–7.31 to 1.37)	0.33
SET vs AC (ref)	–6.71	(–14.02 to 0.47)	(–12.10 to –1.02)	(–10.90 to –2.25)	0.71
Marginal means for change (Time)					
SET	–3.07	(–8.57 to 2.42)			
M2M	0.40	(–5.36 to 6.17)			
AC	3.64	(–1.86 to 9.13)			
**Global Health-Physical, T score**					
Between-group difference for change (time)					
M2M vs AC (ref)	2.47	(–2.94 to 7.31)	(–1.20 to 6.33)	(–0.51 to 5.60)	0.38
SET vs AC (ref)	3.22	(–1.89 to 8.29)	(–0.18 to 7.07)	(0.41-6.44)	0.51
Marginal means for change (time)					
SET	2.25	(–1.50 to 6.00)			
M2M	1.49	(–2.47 to 5.45)			
AC	–0.97	(–4.55 to 2.61)			
**Global Health-Mental, T score**					
Between-group difference for change (time)					
M2M vs AC (ref)	–1.99	(–8.13 to 3.23)	(–6.30 to 2.43)	(–5.27 to 1.47)	0.27
SET vs AC (ref)	0.01	(–5.99 to 5.04)	(–3.85 to 3.97)	(–3.63 to 3.39)	0.00019
Marginal means for change (time)					
SET	0.66	(–3.55 to 4.87)			
M2M	–1.35	(–5.79 to 3.09)			
AC	0.65	(–3.36 to 4.66)			
**Ability to Participate in Social Roles and Activities 8a, T score**					
Between-group difference for change (time)					
M2M vs AC (ref)	–3.18	(–9.66 to 3.24)	(–7.51 to 1.84)	(–6.99 to 0.69)	0.37
SET vs AC (ref)	0.76	(–5.13 to 6.62)	(–3.46 to 5.36)	(–2.88 to 4.53)	0.09
Marginal means for change (time)					
SET	0.69	(–3.98 to 5.37)			
M2M	–3.24	(–8.16 to 1.68)			
AC	–0.06	(–4.53 to 4.41)			

NOTE. CIs for between-group estimates were calculated using the bootstrap method.

Abbreviation: LSM, least square means.

Overall, SET participants had the largest improvements in fatigue, pain intensity, global physical health, and ability to participate in social roles and activities. AC participants reported the largest reduction in pain interference and sleep disturbance but also experienced increased fatigue and a decline in global physical health from baseline to week 8.

## Discussion

The present study assessed the feasibility, platform usability, and acceptability of the SCIPE intervention. A high retention rate of 91.7% during the 8-week intervention suggests that the SCIPE interventions, including SET and M2M, were feasible in maintaining participant engagement. However, attrition increased postintervention, with the follow-up completion rate reduced to 58.3% by week 16. This decline in retention is not uncommon in longitudinal studies and may reflect challenges in sustaining participant motivation once the structured support of the intervention ends.

Several factors may have contributed to the increased attrition, including participants’ underlying health conditions and the challenges associated with SCI. The self-directed nature of postintervention activities may also have reduced participant engagement. To improve future program iterations, participants suggested increasing opportunities for peer interactions, providing clearer weekly objectives, and incorporating instructional videos demonstrating techniques and adaptations for various injury levels. Specific requests included exercises targeting particular muscle groups, more advanced options, use of upbeat music, and videos featuring both instructors and participants to better illustrate adaptations. One participant also suggested adding a pause feature to improve the overall user experience. Further investigation is needed to identify strategies for improving long-term retention, such as sustained engagement efforts and additional postintervention support.

The overall usability of the SCIPE platform, as assessed by the SUS, was rated as “fair.” While participants generally found the platform usable, there was considerable room for improvement. Suggested enhancements included better reminder features, the ability to track actual workouts alongside completed videos and articles, graphical displays of personal and collective progress for accountability, and simplifying content to reduce the number of web pages. Additionally, SET participants rated the platform’s usability as “good” while AC and M2M participants rated it as “fair,” suggesting that the structure or content of the SET intervention platform may have facilitated a more positive user experience.

The overall score from the Health-ITUES outcomes further suggested moderate participant satisfaction with the SCIPE platform. The *Effect* subscale was scored the highest, indicating that participants felt the platform had a positive influence on their health-related outcomes. On the other hand, the *Perceived Usefulness* subscale had the lowest scores, suggesting that participants did not find the platform as practical or applicable to their daily lives as hoped. Findings from the Health-ITUES presents an area for improvement, as increasing the platform’s perceived usefulness could enhance participant engagement and adherence.

Given the exploratory nature of this interim analysis, our primary focus was to estimate effect sizes for the physical activity and quality of life outcomes. Overall, SET participants demonstrated the largest improvements in mild, moderate, and total physical activity from baseline to week 8, while M2M participants showed the smallest changes across all categories. Interestingly, vigorous activity decreased in SET participants, indicating a moderate negative intervention effect. In contrast, AC participants had unexpectedly large increases in physical activity, particularly in the vigorous category, suggesting the presence of nonspecific effects such as increased attention from study staff, engagement in health-related goal setting, or enhanced motivation from study participation. In terms of quality of life outcomes, SET participants reported moderate improvements in fatigue and global physical health, while other PROMIS measures showed small to negligible effects. The M2M participants showed no significant changes across PROMIS measures, while AC participants reported reduced pain interference and sleep disturbance but increased fatigue and declines in global physical health. These findings highlight the complexity of behavior change and the importance of including AC groups in exercise trials. While the current study was not powered to detect statistical significance, the observed trends provide valuable insight into the potential of remote interventions for people with SCI. Larger, fully powered trials are needed to confirm these findings and better understand the mechanisms driving changes in health outcomes.

### Study limitations

The study has several limitations. As an interim analysis, the primary aims were to evaluate the feasibility, usability, and acceptability of the SCIPE interventions and to obtain preliminary effect size estimates for the study outcomes. Thus, the findings should be interpreted with caution because of the small sample size. Additionally, because the interventions were delivered asynchronously, it was difficult to verify whether participants consistently followed the exercises or engaged with the articles. Long-term participant engagement after the intervention also presented a challenge, highlighting the need for future studies to identify factors contributing to attrition and to explore strategies for enhancing sustained engagement.

## Conclusions

The SCIPE study demonstrated high feasibility during the intervention period but faced challenges with long-term participant retention. While the SCIPE platform demonstrated moderate usability and acceptability, there remains substantial room for improvement, particularly in enhancing perceived usefulness and sustaining engagement beyond the intervention. Future studies should involve larger sample sizes to validate these preliminary findings and draw more robust conclusions. Additionally, refining the platform’s design and functionality, alongside exploring strategies to promote long-term retention, will be crucial for improving the effectiveness and acceptability of similar interventions for people with SCI.

## Supplier

a. R, version 4.3; R Core Team.

## Disclosure

The investigators have no financial or nonfinancial disclosures to make in relation to this project.

## Appendix Supplementary materials

Image, application 1

## Footnotes

Supplementary material associated with this article can be found, in the online version, at doi:10.1016/j.arrct.2025.100495.
