# Supplementary material for: Feasibility, Usability, and Acceptability of a Randomized Controlled Trial Evaluating Teleexercise Interventions for Individuals with Spinal Cord Injury: Interim Analysis of the Spinal Cord Injury Program in Exercise (SCIPE) Study
Source: Arch Rehabil Res Clin Transl. 2025 Jul 17;7(3):100495. doi: 10.1016/j.arrct.2025.100495 (PMC12447203; doi:10.1016/j.arrct.2025.100495)
Supplement: Supplementary file 1 [file mmc1.docx]

**SCIPE Intervention Class Content and Fidelity Checklists**

2018-2023

**Standard Exercise Training (SET) Intervention**

| **SET Week 1** | | **Four 8-count breathing cycles at the beginning** | | | | | |
| --- | --- | --- | --- | --- | --- | --- | --- |
|  |  | **Duration**  **(minute)** | **Body Part** | **Movement** | **Pattern** | **Time**  **(sec)** | **Check** |
| **Range of Motion** | **Upper Body** | 5 | Hand | Extension | Static | 10 |  |
|  |  |  |  | Flexion | Static |  |  |
|  |  |  | Wrist | Extension | Static |  |  |
|  |  |  |  | Flexion | Static |  |  |
|  |  |  |  | Adduction | Static |  |  |
|  |  |  |  | Abduction | Static |  |  |
|  |  |  |  | Circumduction | Static |  |  |
|  |  |  | Elbow | Extension | Static | 10 |  |
|  |  |  |  | Flexion | Static |  |  |
|  |  |  | Shoulder | Extension | Static | 15 |  |
|  |  |  |  | Flexion | Static |  |  |
|  |  |  |  | Adduction | Static |  |  |
|  |  |  |  | Abduction | Static |  |  |
|  |  |  |  | Horizontal adduction | Static |  |  |
|  |  |  |  | Horizontal abduction | Static |  |  |
|  |  |  |  | Shoulder girdle elevation/depression | Static |  |  |
|  |  |  |  | Rotation (without arms) | Static |  |  |
|  |  |  | Neck | Extension | Static | 15 |  |
|  |  |  |  | Flexion | Static |  |  |
|  |  |  |  | Lateral extension | Static |  |  |
|  |  |  |  | Rotation | Static |  |  |
|  |  |  | Torso | Fontal extension | Static | 15 |  |
|  |  |  |  | Dorsal extension | Static |  |  |
|  |  |  |  | Lateral extension | Static |  |  |
|  |  |  |  | Rotation | Static |  |  |

| **SET Week 2** | | **Four 8-count breathing cycles at the beginning** | | | | | |
| --- | --- | --- | --- | --- | --- | --- | --- |
|  |  | **Duration**  **(minute)** | **Body Part** | **Movement** | **Pattern** | **Time**  **(sec)** | **Check** |
| **Range of Motion** | **Upper Body** | 5 | Hand | Extension | Static | 10 |  |
|  |  |  |  | Flexion | Static |  |  |
|  |  |  | Wrist | Extension | Static |  |  |
|  |  |  |  | Flexion | Static |  |  |
|  |  |  |  | Adduction | Static |  |  |
|  |  |  |  | Abduction | Static |  |  |
|  |  |  |  | Circumduction | Static |  |  |
|  |  |  | Elbow | Extension | Static | 10 |  |
|  |  |  |  | Flexion | Static |  |  |
|  |  |  | Shoulder | Extension | Static | 15 |  |
|  |  |  |  | Flexion | Static |  |  |
|  |  |  |  | Adduction | Static |  |  |
|  |  |  |  | Abduction | Static |  |  |
|  |  |  |  | Horizontal adduction | Static |  |  |
|  |  |  |  | Horizontal abduction | Static |  |  |
|  |  |  |  | Shoulder girdle elevation/depression | Static |  |  |
|  |  |  |  | Rotation (without arms) | Static |  |  |
|  |  |  | Neck | Extension | Static | 15 |  |
|  |  |  |  | Flexion | Static |  |  |
|  |  |  |  | Lateral extension | Static |  |  |
|  |  |  |  | Rotation | Static |  |  |
|  |  |  | Torso | Fontal extension | Static | 15 |  |
|  |  |  |  | Dorsal extension | Static |  |  |
|  |  |  |  | Lateral extension | Static |  |  |
|  |  |  |  | Rotation | Static |  |  |
|  | **Lower Body** | 5 | Hip | Flexion | Static | 15 |  |
|  |  |  |  | Extension | Static |  |  |
|  |  |  |  | Adduction | Static |  |  |
|  |  |  |  | Abduction | Static |  |  |
|  |  |  |  | Internal rotation | Static |  |  |
|  |  |  |  | External rotation | Static |  |  |
|  |  |  | Knee | Extension | Static | 15 |  |
|  |  |  |  | Flexion | Static |  |  |
|  |  |  | Ankle | Extension | Static | 10 |  |
|  |  |  |  | Flexion | Static |  |  |
|  |  |  |  | Circumduction | Static |  |  |
|  |  |  | Toes | Flexion | Static | 10 |  |
|  |  |  |  | Extension | Static |  |  |

| **SET Week 3** | | **Four 8-count breathing cycles at the beginning** | | | | | |
| --- | --- | --- | --- | --- | --- | --- | --- |
|  |  | **Duration**  **(minute)** | **Body Part** | **Movement** | **Pattern** | **Time**  **(sec)** | **Check** |
| **Range of Motion** | **Upper Body** | 5 | Hand | Extension | Static | 10 |  |
|  |  |  |  | Flexion | Static |  |  |
|  |  |  | Wrist | Extension | Static |  |  |
|  |  |  |  | Flexion | Static |  |  |
|  |  |  |  | Adduction | Static |  |  |
|  |  |  |  | Abduction | Static |  |  |
|  |  |  |  | Circumduction | Static |  |  |
|  |  |  | Elbow | Extension | Static | 10 |  |
|  |  |  |  | Flexion | Static |  |  |
|  |  |  | Shoulder | Extension | Static | 15 |  |
|  |  |  |  | Flexion | Static |  |  |
|  |  |  |  | Adduction | Static |  |  |
|  |  |  |  | Abduction | Static |  |  |
|  |  |  |  | Horizontal adduction | Static |  |  |
|  |  |  |  | Horizontal abduction | Static |  |  |
|  |  |  |  | Shoulder girdle elevation/depression | Static |  |  |
|  |  |  |  | Rotation (without arms) | Static |  |  |
|  |  |  | Neck | Extension | Static | 15 |  |
|  |  |  |  | Flexion | Static |  |  |
|  |  |  |  | Lateral extension | Static |  |  |
|  |  |  |  | Rotation | Static |  |  |
|  |  |  | Torso | Fontal extension | Static | 15 |  |
|  |  |  |  | Dorsal extension | Static |  |  |
|  |  |  |  | Lateral extension | Static |  |  |
|  |  |  |  | Rotation | Static |  |  |
|  | **Lower Body** | 5 | Hip | Flexion | Static | 15 |  |
|  |  |  |  | Extension | Static |  |  |
|  |  |  |  | Adduction | Static |  |  |
|  |  |  |  | Abduction | Static |  |  |
|  |  |  |  | Internal rotation | Static |  |  |
|  |  |  |  | External rotation | Static |  |  |
|  |  |  | Knee | Extension | Static | 15 |  |
|  |  |  |  | Flexion | Static |  |  |
|  |  |  | Ankle | Extension | Static | 10 |  |
|  |  |  |  | Flexion | Static |  |  |
|  |  |  |  | Circumduction | Static |  |  |
|  |  |  | Toes | Flexion | Static | 10 |  |
|  |  |  |  | Extension | Static |  |  |

| **SET Week 3** | | **Duration**  **(minute)** | **Body Part** | **Muscle** | **Repetition** | **Set** | **Contraction/Muscle** | **Pattern** | **Tempo**  **(bpm)** | **Check** |
| --- | --- | --- | --- | --- | --- | --- | --- | --- | --- | --- |
| **Strength** | **Upper Body** | 5 | Shoulder | Deltoids | 8 | 1 | 8 | Linear | 40-60 |  |
|  |  |  | Chest | Trapezius/Rhomboids | 8 | 1 | 8 | Linear | 40-60 |  |
|  |  |  |  | Pectorals | 8 | 1 | 8 | Linear | 40-60 |  |
|  |  |  | Arm | Biceps | 8 | 1 | 8 | Linear | 40-80 |  |
|  |  |  |  | Triceps | 8 | 1 | 8 | Linear | 40-80 |  |
|  | **Lower Body** |  | Trunk | Erector Spinae | 8 | 1 | 8 | Linear | 40-60 |  |
|  |  |  |  | Rectus abdominus | 8 | 1 | 8 | Linear | 40-80 |  |
|  |  |  |  | Obliques | 8 | 1 | 8 | Linear | 40-80 |  |

| **SET Week 3** | **Duration**  **(minute)** | **Movement** | **Pattern** | **Repetition** | **Set** | **Contraction/Movement** | **Check** |
| --- | --- | --- | --- | --- | --- | --- | --- |
| **Functional Strength**  **/Balance** | 5 | Squats | Feet parallel | 8 | 1 | 8 |  |
|  |  | Calf Raises | Feet parallel | 8 | 1 | 8 |  |
|  |  | Leg Lifts | Front, side and back | 8/direction | 1 | 8/direction |  |
|  |  | Static Balance | One leg balance |  |  |  |  |

| **SET Week 4** | | **Four 8-count breathing cycles at the beginning** | | | | | |
| --- | --- | --- | --- | --- | --- | --- | --- |
|  |  | **Duration**  **(minute)** | **Body Part** | **Movement** | **Pattern** | **Time**  **(sec)** | **Check** |
| **Range of Motion** | **Upper Body** | 5 | Hand | Extension | Static | 10 |  |
|  |  |  |  | Flexion | Static |  |  |
|  |  |  | Wrist | Extension | Static |  |  |
|  |  |  |  | Flexion | Static |  |  |
|  |  |  |  | Adduction | Static |  |  |
|  |  |  |  | Abduction | Static |  |  |
|  |  |  |  | Circumduction | Static |  |  |
|  |  |  | Elbow | Extension | Static | 10 |  |
|  |  |  |  | Flexion | Static |  |  |
|  |  |  | Shoulder | Extension | Static | 15 |  |
|  |  |  |  | Flexion | Static |  |  |
|  |  |  |  | Adduction | Static |  |  |
|  |  |  |  | Abduction | Static |  |  |
|  |  |  |  | Horizontal adduction | Static |  |  |
|  |  |  |  | Horizontal abduction | Static |  |  |
|  |  |  |  | Shoulder girdle elevation/depression | Static |  |  |
|  |  |  |  | Rotation (without arms) | Static |  |  |
|  |  |  | Neck | Extension | Static | 15 |  |
|  |  |  |  | Flexion | Static |  |  |
|  |  |  |  | Lateral extension | Static |  |  |
|  |  |  |  | Rotation | Static |  |  |
|  |  |  | Torso | Fontal extension | Static | 15 |  |
|  |  |  |  | Dorsal extension | Static |  |  |
|  |  |  |  | Lateral extension | Static |  |  |
|  |  |  |  | Rotation | Static |  |  |
|  | **Lower Body** | 5 | Hip | Flexion | Static | 15 |  |
|  |  |  |  | Extension | Static |  |  |
|  |  |  |  | Adduction | Static |  |  |
|  |  |  |  | Abduction | Static |  |  |
|  |  |  |  | Internal rotation | Static |  |  |
|  |  |  |  | External rotation | Static |  |  |
|  |  |  | Knee | Extension | Static | 15 |  |
|  |  |  |  | Flexion | Static |  |  |
|  |  |  | Ankle | Extension | Static | 10 |  |
|  |  |  |  | Flexion | Static |  |  |
|  |  |  |  | Circumduction | Static |  |  |
|  |  |  | Toes | Flexion | Static | 10 |  |
|  |  |  |  | Extension | Static |  |  |

| **SET Week 4** | | **Duration**  **(minute)** | **Body Part** | **Muscle** | **Repetition** | **Set** | **Contraction/Muscle** | **Pattern** | **Tempo**  **(bpm)** | **Check** |
| --- | --- | --- | --- | --- | --- | --- | --- | --- | --- | --- |
| **Strength** | **Upper Body** | 5 | Shoulder | Deltoids | 8 | 1 | 8 | Linear | 40-60 |  |
|  |  |  | Chest | Trapezius/Rhomboids | 8 | 1 | 8 | Linear | 40-60 |  |
|  |  |  |  | Pectorals | 8 | 1 | 8 | Linear | 40-60 |  |
|  |  |  | Arm | Biceps | 8 | 1 | 8 | Linear | 40-80 |  |
|  |  |  |  | Triceps | 8 | 1 | 8 | Linear | 40-80 |  |
|  | **Lower Body** |  | Trunk | Erector Spinae | 8 | 1 | 8 | Linear | 40-60 |  |
|  |  |  |  | Rectus abdominus | 8 | 1 | 8 | Linear | 40-80 |  |
|  |  |  |  | Obliques | 8 | 1 | 8 | Linear | 40-80 |  |

| **SET Week 4** | **Time (minute)** | **Movement** | **Repetition** | **Set** | **Position** | **Pattern** | **Equipment** | **Tempo (movement)** | **Check** |
| --- | --- | --- | --- | --- | --- | --- | --- | --- | --- |
| **Cardio** | 5 | Any | - | - | Sitting | Constant move | Chair | 100-180 |  |

| **SET Week 4** | **Duration**  **(minute)** | **Movement** | **Pattern** | **Repetition** | **Set** | **Contraction/Movement** | **Check** |
| --- | --- | --- | --- | --- | --- | --- | --- |
| **Functional Strength**  **/Balance** | 5 | Squats | Feet parallel | 8 | 1 | 8 |  |
|  |  | Calf Raises | Feet parallel | 8 | 1 | 8 |  |
|  |  | Leg Lifts | Front, side and back | 8/direction | 1 | 8/direction |  |
|  |  | Static Balance | One leg balance |  |  |  |  |

| **SET Week 4** | **Duration (minute)** | **Movement** | **Pattern** | **Repetition** | **Set** | **Position** | **Check** |
| --- | --- | --- | --- | --- | --- | --- | --- |
| **Cool Down** | 5 | Lateral breathing | Linear | - | - | Standing  /Sitting |  |
|  |  | Breathing with arm stretch | Linear | - | - | Standing  /Sitting |  |
|  |  | Breathing with torso stretch | Linear | - | - | Standing  /Sitting |  |
|  |  | Breathing with leg stretch | Linear | - | - | Standing  /Sitting |  |
|  |  | Breathing with hand stretch | Linear | - | - | Standing  /Sitting |  |
|  |  | Breathing with neck stretch | Linear | - | - | Standing  /Sitting |  |

| **SET Week 5** | | **Four 8-count breathing cycles at the beginning** | | | | | |
| --- | --- | --- | --- | --- | --- | --- | --- |
|  |  | **Duration**  **(minute)** | **Body Part** | **Movement** | **Pattern** | **Time**  **(sec)** | **Check** |
| **Range of Motion** | **Upper Body** | 5 | Hand | Extension | Static | 10 |  |
|  |  |  |  | Flexion | Static |  |  |
|  |  |  | Wrist | Extension | Static |  |  |
|  |  |  |  | Flexion | Static |  |  |
|  |  |  |  | Adduction | Static |  |  |
|  |  |  |  | Abduction | Static |  |  |
|  |  |  |  | Circumduction | Static |  |  |
|  |  |  | Elbow | Extension | Static | 10 |  |
|  |  |  |  | Flexion | Static |  |  |
|  |  |  | Shoulder | Extension | Static | 15 |  |
|  |  |  |  | Flexion | Static |  |  |
|  |  |  |  | Adduction | Static |  |  |
|  |  |  |  | Abduction | Static |  |  |
|  |  |  |  | Horizontal adduction | Static |  |  |
|  |  |  |  | Horizontal abduction | Static |  |  |
|  |  |  |  | Shoulder girdle elevation/depression | Static |  |  |
|  |  |  |  | Rotation (without arms) | Static |  |  |
|  |  |  | Neck | Extension | Static | 15 |  |
|  |  |  |  | Flexion | Static |  |  |
|  |  |  |  | Lateral extension | Static |  |  |
|  |  |  |  | Rotation | Static |  |  |
|  |  |  | Torso | Fontal extension | Static | 15 |  |
|  |  |  |  | Dorsal extension | Static |  |  |
|  |  |  |  | Lateral extension | Static |  |  |
|  |  |  |  | Rotation | Static |  |  |
|  | **Lower Body** | 5 | Hip | Flexion | Static | 15 |  |
|  |  |  |  | Extension | Static |  |  |
|  |  |  |  | Adduction | Static |  |  |
|  |  |  |  | Abduction | Static |  |  |
|  |  |  |  | Internal rotation | Static |  |  |
|  |  |  |  | External rotation | Static |  |  |
|  |  |  | Knee | Extension | Static | 15 |  |
|  |  |  |  | Flexion | Static |  |  |
|  |  |  | Ankle | Extension | Static | 10 |  |
|  |  |  |  | Flexion | Static |  |  |
|  |  |  |  | Circumduction | Static |  |  |
|  |  |  | Toes | Flexion | Static | 10 |  |
|  |  |  |  | Extension | Static |  |  |

| **SET Week 5** | | **Duration**  **(minute)** | **Body Part** | **Muscle** | **Repetition** | **Set** | **Contraction/Muscle** | **Pattern** | **Tempo**  **(bpm)** | **Check** |
| --- | --- | --- | --- | --- | --- | --- | --- | --- | --- | --- |
| **Strength** | **Upper Body** | 5 | Shoulder | Deltoids | 10 | 1 | 10 | Linear | 40-60 |  |
|  |  |  | Chest | Trapezius/Rhomboids | 10 | 1 | 10 | Linear | 40-60 |  |
|  |  |  |  | Pectorals | 10 | 1 | 10 | Linear | 40-60 |  |
|  |  |  | Arm | Biceps | 10 | 1 | 10 | Linear | 40-80 |  |
|  |  |  |  | Triceps | 10 | 1 | 10 | Linear | 40-80 |  |
|  | **Lower Body** |  | Trunk | Erector Spinae | 10 | 1 | 10 | Linear | 40-60 |  |
|  |  |  |  | Rectus abdominus | 10 | 1 | 10 | Linear | 40-80 |  |
|  |  |  |  | Obliques | 10 | 1 | 10 | Linear | 40-80 |  |

| **SET Week 5** | **Time (minute)** | **Movement** | **Repetition** | **Set** | **Position** | **Pattern** | **Equipment** | **Tempo (movement)** | **Check** |
| --- | --- | --- | --- | --- | --- | --- | --- | --- | --- |
| **Cardio** | 5 | Any | - | - | Sitting | Constant move | Chair | 100-180 |  |

| **SET Week 5** | **Duration**  **(minute)** | **Movement** | **Pattern** | **Repetition** | **Set** | **Contraction/Movement** | **Check** |
| --- | --- | --- | --- | --- | --- | --- | --- |
| **Functional Strength**  **/Balance** | 5 | Squats | Feet parallel | 8 | 1 | 8 |  |
|  |  | Calf Raises | Feet parallel | 8 | 1 | 8 |  |
|  |  | Leg Lifts | Front, side and back | 8/direction | 1 | 8/direction |  |
|  |  | Weight Shifting | 3 directions (front, side and back) both legs; keep supporting leg on the ground |  |  |  |  |
|  |  | Static Balance | One leg balance |  |  |  |  |

| **SET Week 5** | **Duration (minute)** | **Movement** | **Pattern** | **Repetition** | **Set** | **Position** | **Check** |
| --- | --- | --- | --- | --- | --- | --- | --- |
| **Cool Down** | 5 | Lateral breathing | Linear | - | - | Standing  /Sitting |  |
|  |  | Breathing with arm stretch | Linear | - | - | Standing  /Sitting |  |
|  |  | Breathing with torso stretch | Linear | - | - | Standing  /Sitting |  |
|  |  | Breathing with leg stretch | Linear | - | - | Standing  /Sitting |  |
|  |  | Breathing with hand stretch | Linear | - | - | Standing  /Sitting |  |
|  |  | Breathing with neck stretch | Linear | - | - | Standing  /Sitting |  |

| **SET Week 6** | | **Four 8-count breathing cycles at the beginning** | | | | | |
| --- | --- | --- | --- | --- | --- | --- | --- |
|  |  | **Duration**  **(minute)** | **Body Part** | **Movement** | **Pattern** | **Time**  **(sec)** | **Check** |
| **Range of Motion** | **Upper Body** | 5 | Hand | Extension | Static | 10 |  |
|  |  |  |  | Flexion | Static |  |  |
|  |  |  | Wrist | Extension | Static |  |  |
|  |  |  |  | Flexion | Static |  |  |
|  |  |  |  | Adduction | Static |  |  |
|  |  |  |  | Abduction | Static |  |  |
|  |  |  |  | Circumduction | Static |  |  |
|  |  |  | Elbow | Extension | Static | 10 |  |
|  |  |  |  | Flexion | Static |  |  |
|  |  |  | Shoulder | Extension | Static | 15 |  |
|  |  |  |  | Flexion | Static |  |  |
|  |  |  |  | Adduction | Static |  |  |
|  |  |  |  | Abduction | Static |  |  |
|  |  |  |  | Horizontal adduction | Static |  |  |
|  |  |  |  | Horizontal abduction | Static |  |  |
|  |  |  |  | Shoulder girdle elevation/depression | Static |  |  |
|  |  |  |  | Rotation (without arms) | Static |  |  |
|  |  |  | Neck | Extension | Static | 15 |  |
|  |  |  |  | Flexion | Static |  |  |
|  |  |  |  | Lateral extension | Static |  |  |
|  |  |  |  | Rotation | Static |  |  |
|  |  |  | Torso | Fontal extension | Static | 15 |  |
|  |  |  |  | Dorsal extension | Static |  |  |
|  |  |  |  | Lateral extension | Static |  |  |
|  |  |  |  | Rotation | Static |  |  |
|  | **Lower Body** | 5 | Hip | Flexion | Static | 15 |  |
|  |  |  |  | Extension | Static |  |  |
|  |  |  |  | Adduction | Static |  |  |
|  |  |  |  | Abduction | Static |  |  |
|  |  |  |  | Internal rotation | Static |  |  |
|  |  |  |  | External rotation | Static |  |  |
|  |  |  | Knee | Extension | Static | 15 |  |
|  |  |  |  | Flexion | Static |  |  |
|  |  |  | Ankle | Extension | Static | 10 |  |
|  |  |  |  | Flexion | Static |  |  |
|  |  |  |  | Circumduction | Static |  |  |
|  |  |  | Toes | Flexion | Static | 10 |  |
|  |  |  |  | Extension | Static |  |  |

| **SET Week 6** | | **Duration**  **(minute)** | **Body Part** | **Muscle** | **Repetition** | **Set** | **Contraction/Muscle** | **Pattern** | **Tempo**  **(bpm)** | **Check** |
| --- | --- | --- | --- | --- | --- | --- | --- | --- | --- | --- |
| **Strength** | **Upper Body** | 5 | Shoulder | Deltoids | 10 | 1 | 10 | Linear | 40-60 |  |
|  |  |  | Chest | Trapezius/Rhomboids | 10 | 1 | 10 | Linear | 40-60 |  |
|  |  |  |  | Pectorals | 10 | 1 | 10 | Linear | 40-60 |  |
|  |  |  | Arm | Biceps | 10 | 1 | 10 | Linear | 40-80 |  |
|  |  |  |  | Triceps | 10 | 1 | 10 | Linear | 40-80 |  |
|  | **Lower Body** |  | Trunk | Erector Spinae | 10 | 1 | 10 | Linear | 40-60 |  |
|  |  |  |  | Rectus abdominus | 10 | 1 | 10 | Linear | 40-80 |  |
|  |  |  |  | Obliques | 10 | 1 | 10 | Linear | 40-80 |  |

| **SET Week 6** | **Time (minute)** | **Movement** | **Repetition** | **Set** | **Position** | **Pattern** | **Equipment** | **Tempo (movement)** | **Check** |
| --- | --- | --- | --- | --- | --- | --- | --- | --- | --- |
| **Cardio** | 15 | Any | - | - | Sitting | Constant move | Chair | 100-180 |  |

| **SET Week 6** | **Duration**  **(minute)** | **Movement** | **Pattern** | **Repetition** | **Set** | **Contraction/Movement** | **Check** |
| --- | --- | --- | --- | --- | --- | --- | --- |
| **Functional Strength**  **/Balance** | 5 | Squats | Feet parallel | 8 | 1 | 8 |  |
|  |  | Calf Raises | Feet parallel | 8 | 1 | 8 |  |
|  |  | Leg Lifts | Front, side and back | 8/direction | 1 | 8/direction |  |
|  |  | Weight Shifting | 3 directions (front, side and back) both legs; keep supporting leg on the ground |  |  |  |  |
|  |  | Static Balance | One leg balance |  |  |  |  |

| **SET Week 6** | **Duration (minute)** | **Movement** | **Pattern** | **Repetition** | **Set** | **Position** | **Check** |
| --- | --- | --- | --- | --- | --- | --- | --- |
| **Cool Down** | 5 | Lateral breathing | Linear | - | - | Standing  /Sitting |  |
|  |  | Breathing with arm stretch | Linear | - | - | Standing  /Sitting |  |
|  |  | Breathing with torso stretch | Linear | - | - | Standing  /Sitting |  |
|  |  | Breathing with leg stretch | Linear | - | - | Standing  /Sitting |  |
|  |  | Breathing with hand stretch | Linear | - | - | Standing  /Sitting |  |
|  |  | Breathing with neck stretch | Linear | - | - | Standing  /Sitting |  |

| **SET Week 7** | | **Four 8-count breathing cycles at the beginning** | | | | | |
| --- | --- | --- | --- | --- | --- | --- | --- |
|  |  | **Duration**  **(minute)** | **Body Part** | **Movement** | **Pattern** | **Time**  **(sec)** | **Check** |
| **Range of Motion** | **Upper Body** | 5 | Hand | Extension | Static | 10 |  |
|  |  |  |  | Flexion | Static |  |  |
|  |  |  | Wrist | Extension | Static |  |  |
|  |  |  |  | Flexion | Static |  |  |
|  |  |  |  | Adduction | Static |  |  |
|  |  |  |  | Abduction | Static |  |  |
|  |  |  |  | Circumduction | Static |  |  |
|  |  |  | Elbow | Extension | Static | 10 |  |
|  |  |  |  | Flexion | Static |  |  |
|  |  |  | Shoulder | Extension | Static | 15 |  |
|  |  |  |  | Flexion | Static |  |  |
|  |  |  |  | Adduction | Static |  |  |
|  |  |  |  | Abduction | Static |  |  |
|  |  |  |  | Horizontal adduction | Static |  |  |
|  |  |  |  | Horizontal abduction | Static |  |  |
|  |  |  |  | Shoulder girdle elevation/depression | Static |  |  |
|  |  |  |  | Rotation (without arms) | Static |  |  |
|  |  |  | Neck | Extension | Static | 15 |  |
|  |  |  |  | Flexion | Static |  |  |
|  |  |  |  | Lateral extension | Static |  |  |
|  |  |  |  | Rotation | Static |  |  |
|  |  |  | Torso | Fontal extension | Static | 15 |  |
|  |  |  |  | Dorsal extension | Static |  |  |
|  |  |  |  | Lateral extension | Static |  |  |
|  |  |  |  | Rotation | Static |  |  |
|  | **Lower Body** | 5 | Hip | Flexion | Static | 15 |  |
|  |  |  |  | Extension | Static |  |  |
|  |  |  |  | Adduction | Static |  |  |
|  |  |  |  | Abduction | Static |  |  |
|  |  |  |  | Internal rotation | Static |  |  |
|  |  |  |  | External rotation | Static |  |  |
|  |  |  | Knee | Extension | Static | 15 |  |
|  |  |  |  | Flexion | Static |  |  |
|  |  |  | Ankle | Extension | Static | 10 |  |
|  |  |  |  | Flexion | Static |  |  |
|  |  |  |  | Circumduction | Static |  |  |
|  |  |  | Toes | Flexion | Static | 10 |  |
|  |  |  |  | Extension | Static |  |  |

| **SET Week 7** | | **Duration**  **(minute)** | **Body Part** | **Muscle** | **Repetition** | **Set** | **Contraction/Muscle** | **Pattern** | **Tempo**  **(bpm)** | **Check** |
| --- | --- | --- | --- | --- | --- | --- | --- | --- | --- | --- |
| **Strength** | **Upper Body** | 5 | Shoulder | Deltoids | 12 | 1 | 12 | Linear | 40-60 |  |
|  |  |  | Chest | Trapezius/Rhomboids | 12 | 1 | 12 | Linear | 40-60 |  |
|  |  |  |  | Pectorals | 12 | 1 | 12 | Linear | 40-60 |  |
|  |  |  | Arm | Biceps | 12 | 1 | 12 | Linear | 40-80 |  |
|  |  |  |  | Triceps | 12 | 1 | 12 | Linear | 40-80 |  |
|  | **Lower Body** |  | Trunk | Erector Spinae | 12 | 1 | 12 | Linear | 40-60 |  |
|  |  |  |  | Rectus abdominus | 12 | 1 | 12 | Linear | 40-80 |  |
|  |  |  |  | Obliques | 12 | 1 | 12 | Linear | 40-80 |  |

| **SET Week 7** | **Time (minute)** | **Movement** | **Repetition** | **Set** | **Position** | **Pattern** | **Equipment** | **Tempo (movement)** | **Check** |
| --- | --- | --- | --- | --- | --- | --- | --- | --- | --- |
| **Cardio** | 15 | Any | - | - | Sitting | Constant move | Chair | 100-180 |  |

| **SET Week 7** | **Duration**  **(minute)** | **Movement** | **Pattern** | **Repetition** | **Set** | **Contraction/Movement** | **Check** |
| --- | --- | --- | --- | --- | --- | --- | --- |
| **Functional Strength**  **/Balance** | 5 | Squats | Feet parallel | 8 | 1 | 8 |  |
|  |  | Calf Raises | Feet parallel | 8 | 1 | 8 |  |
|  |  | Leg Lifts | Front, side and back | 8/direction | 1 | 8/direction |  |
|  |  | Weight Shifting | 3 directions (front, side, back and diagonal) both legs; bring supporting leg off ground |  |  |  |  |

| **SET Week 7** | **Duration (minute)** | **Movement** | **Pattern** | **Repetition** | **Set** | **Position** | **Check** |
| --- | --- | --- | --- | --- | --- | --- | --- |
| **Cool Down** | 5 | Lateral breathing | Linear | - | - | Standing  /Sitting |  |
|  |  | Breathing with arm stretch | Linear | - | - | Standing  /Sitting |  |
|  |  | Breathing with torso stretch | Linear | - | - | Standing  /Sitting |  |
|  |  | Breathing with leg stretch | Linear | - | - | Standing  /Sitting |  |
|  |  | Breathing with hand stretch | Linear | - | - | Standing  /Sitting |  |
|  |  | Breathing with neck stretch | Linear | - | - | Standing  /Sitting |  |

| **SET Week 8** | | **Four 8-count breathing cycles at the beginning** | | | | | |
| --- | --- | --- | --- | --- | --- | --- | --- |
|  |  | **Duration**  **(minute)** | **Body Part** | **Movement** | **Pattern** | **Time**  **(sec)** | **Check** |
| **Range of Motion** | **Upper Body** | 5 | Hand | Extension | Static | 10 |  |
|  |  |  |  | Flexion | Static |  |  |
|  |  |  | Wrist | Extension | Static |  |  |
|  |  |  |  | Flexion | Static |  |  |
|  |  |  |  | Adduction | Static |  |  |
|  |  |  |  | Abduction | Static |  |  |
|  |  |  |  | Circumduction | Static |  |  |
|  |  |  | Elbow | Extension | Static | 10 |  |
|  |  |  |  | Flexion | Static |  |  |
|  |  |  | Shoulder | Extension | Static | 15 |  |
|  |  |  |  | Flexion | Static |  |  |
|  |  |  |  | Adduction | Static |  |  |
|  |  |  |  | Abduction | Static |  |  |
|  |  |  |  | Horizontal adduction | Static |  |  |
|  |  |  |  | Horizontal abduction | Static |  |  |
|  |  |  |  | Shoulder girdle elevation/depression | Static |  |  |
|  |  |  |  | Rotation (without arms) | Static |  |  |
|  |  |  | Neck | Extension | Static | 15 |  |
|  |  |  |  | Flexion | Static |  |  |
|  |  |  |  | Lateral extension | Static |  |  |
|  |  |  |  | Rotation | Static |  |  |
|  |  |  | Torso | Fontal extension | Static | 15 |  |
|  |  |  |  | Dorsal extension | Static |  |  |
|  |  |  |  | Lateral extension | Static |  |  |
|  |  |  |  | Rotation | Static |  |  |
|  | **Lower Body** | 5 | Hip | Flexion | Static | 15 |  |
|  |  |  |  | Extension | Static |  |  |
|  |  |  |  | Adduction | Static |  |  |
|  |  |  |  | Abduction | Static |  |  |
|  |  |  |  | Internal rotation | Static |  |  |
|  |  |  |  | External rotation | Static |  |  |
|  |  |  | Knee | Extension | Static | 15 |  |
|  |  |  |  | Flexion | Static |  |  |
|  |  |  | Ankle | Extension | Static | 10 |  |
|  |  |  |  | Flexion | Static |  |  |
|  |  |  |  | Circumduction | Static |  |  |
|  |  |  | Toes | Flexion | Static | 10 |  |
|  |  |  |  | Extension | Static |  |  |

| **SET Week 8** | | **Duration**  **(minute)** | **Body Part** | **Muscle** | **Repetition** | **Set** | **Contraction/Muscle** | **Pattern** | **Tempo**  **(bpm)** | **Check** |
| --- | --- | --- | --- | --- | --- | --- | --- | --- | --- | --- |
| **Strength** | **Upper Body** | 5 | Shoulder | Deltoids | 12 | 1 | 12 | Linear | 40-60 |  |
|  |  |  | Chest | Trapezius/Rhomboids | 12 | 1 | 12 | Linear | 40-60 |  |
|  |  |  |  | Pectorals | 12 | 1 | 12 | Linear | 40-60 |  |
|  |  |  | Arm | Biceps | 12 | 1 | 12 | Linear | 40-80 |  |
|  |  |  |  | Triceps | 12 | 1 | 12 | Linear | 40-80 |  |
|  | **Lower Body** |  | Trunk | Erector Spinae | 12 | 1 | 12 | Linear | 40-60 |  |
|  |  |  |  | Rectus abdominus | 12 | 1 | 12 | Linear | 40-80 |  |
|  |  |  |  | Obliques | 12 | 1 | 12 | Linear | 40-80 |  |

| **SET Week 8** | **Time (minute)** | **Movement** | **Repetition** | **Set** | **Position** | **Pattern** | **Equipment** | **Tempo (movement)** | **Check** |
| --- | --- | --- | --- | --- | --- | --- | --- | --- | --- |
| **Cardio** | 15 | Any | - | - | Sitting | Constant move | Chair | 100-180 |  |

| **SET Week 8** | **Duration**  **(minute)** | **Movement** | **Pattern** | **Repetition** | **Set** | **Contraction/Movement** | **Check** |
| --- | --- | --- | --- | --- | --- | --- | --- |
| **Functional Strength**  **/Balance** | 5 | Squats | Feet parallel | 8 | 1 | 8 |  |
|  |  | Calf Raises | Feet parallel | 8 | 1 | 8 |  |
|  |  | Leg Lifts | Front, side and back | 8/direction | 1 | 8/direction |  |
|  |  | Weight Shifting | 3 directions (front, side, back and diagonal) both legs; bring supporting leg off ground |  |  |  |  |

| **SET Week 8** | **Duration (minute)** | **Movement** | **Pattern** | **Repetition** | **Set** | **Position** | **Check** |
| --- | --- | --- | --- | --- | --- | --- | --- |
| **Cool Down** | 5 | Lateral breathing | Linear | - | - | Standing  /Sitting |  |
|  |  | Breathing with arm stretch | Linear | - | - | Standing  /Sitting |  |
|  |  | Breathing with torso stretch | Linear | - | - | Standing  /Sitting |  |
|  |  | Breathing with leg stretch | Linear | - | - | Standing  /Sitting |  |
|  |  | Breathing with hand stretch | Linear | - | - | Standing  /Sitting |  |
|  |  | Breathing with neck stretch | Linear | - | - | Standing  /Sitting |  |

**Movement-to-Music (M2M) Intervention**

| **M2M Week 1** | | **Four 8-count breathing cycles at the beginning** | | | | | |
| --- | --- | --- | --- | --- | --- | --- | --- |
|  |  | **Duration**  **(minute)** | **Body Part** | **Movement** | **Pattern** | **Tempo**  **(bpm)** | **Check** |
| **Range of Motion** | **Upper Body** | 5 | Hand | Extension | Flow | 12-30 |  |
|  |  |  |  | Flexion | Flow |  |  |
|  |  |  | Wrist | Extension | Flow |  |  |
|  |  |  |  | Flexion | Flow |  |  |
|  |  |  |  | Adduction | Flow |  |  |
|  |  |  |  | Abduction | Flow |  |  |
|  |  |  |  | Circumduction | Flow |  |  |
|  |  |  | Elbow | Extension | Flow | 12-30 |  |
|  |  |  |  | Flexion | Flow |  |  |
|  |  |  | Shoulder | Extension | Flow | 12-20 |  |
|  |  |  |  | Flexion | Flow |  |  |
|  |  |  |  | Adduction | Flow |  |  |
|  |  |  |  | Abduction | Flow |  |  |
|  |  |  |  | Horizontal adduction | Flow |  |  |
|  |  |  |  | Horizontal abduction | Flow |  |  |
|  |  |  |  | Shoulder girdle elevation/depression | Flow |  |  |
|  |  |  |  | Rotation (without arms) | Flow |  |  |
|  |  |  | Neck | Extension | Flow | 12-20 |  |
|  |  |  |  | Flexion | Flow |  |  |
|  |  |  |  | Lateral extension | Flow |  |  |
|  |  |  |  | Rotation | Flow |  |  |
|  |  |  | Torso | Fontal extension | Flow | 12-20 |  |
|  |  |  |  | Dorsal extension | Flow |  |  |
|  |  |  |  | Lateral extension | Flow |  |  |
|  |  |  |  | Rotation | Flow |  |  |

| **M2M Week 2** | | **Four 8-count breathing cycles at the beginning** | | | | | |
| --- | --- | --- | --- | --- | --- | --- | --- |
|  |  | **Duration**  **(minute)** | **Body Part** | **Movement** | **Pattern** | **Tempo**  **(bpm)** | **Check** |
| **Range of Motion** | **Upper Body** | 5 | Hand | Extension | Flow | 12-30 |  |
|  |  |  |  | Flexion | Flow |  |  |
|  |  |  | Wrist | Extension | Flow |  |  |
|  |  |  |  | Flexion | Flow |  |  |
|  |  |  |  | Adduction | Flow |  |  |
|  |  |  |  | Abduction | Flow |  |  |
|  |  |  |  | Circumduction | Flow |  |  |
|  |  |  | Elbow | Extension | Flow | 12-30 |  |
|  |  |  |  | Flexion | Flow |  |  |
|  |  |  | Shoulder | Extension | Flow | 12-20 |  |
|  |  |  |  | Flexion | Flow |  |  |
|  |  |  |  | Adduction | Flow |  |  |
|  |  |  |  | Abduction | Flow |  |  |
|  |  |  |  | Horizontal adduction | Flow |  |  |
|  |  |  |  | Horizontal abduction | Flow |  |  |
|  |  |  |  | Shoulder girdle elevation/depression | Flow |  |  |
|  |  |  |  | Rotation (without arms) | Flow |  |  |
|  |  |  | Neck | Extension | Flow | 12-20 |  |
|  |  |  |  | Flexion | Flow |  |  |
|  |  |  |  | Lateral extension | Flow |  |  |
|  |  |  |  | Rotation | Flow |  |  |
|  |  |  | Torso | Fontal extension | Flow | 12-20 |  |
|  |  |  |  | Dorsal extension | Flow |  |  |
|  |  |  |  | Lateral extension | Flow |  |  |
|  |  |  |  | Rotation | Flow |  |  |
|  | **Lower Body** | 5 | Hip | Flexion | Flow | 12-20 |  |
|  |  |  |  | Extension | Flow |  |  |
|  |  |  |  | Adduction | Flow |  |  |
|  |  |  |  | Abduction | Flow |  |  |
|  |  |  |  | Internal rotation | Flow |  |  |
|  |  |  |  | External rotation | Flow |  |  |
|  |  |  | Knee | Extension | Flow | 12-20 |  |
|  |  |  |  | Flexion | Flow |  |  |
|  |  |  | Ankle | Extension | Flow | 12-30 |  |
|  |  |  |  | Flexion | Flow |  |  |
|  |  |  |  | Circumduction | Flow |  |  |
|  |  |  | Toes | Flexion | Flow | 12-30 |  |
|  |  |  |  | Extension | Flow |  |  |

| **M2M Week 3** | | **Four 8-count breathing cycles at the beginning** | | | | | |
| --- | --- | --- | --- | --- | --- | --- | --- |
|  |  | **Duration**  **(minute)** | **Body Part** | **Movement** | **Pattern** | **Tempo**  **(bpm)** | **Check** |
| **Range of Motion** | **Upper Body** | 5 | Hand | Extension | Flow | 12-30 |  |
|  |  |  |  | Flexion | Flow |  |  |
|  |  |  | Wrist | Extension | Flow |  |  |
|  |  |  |  | Flexion | Flow |  |  |
|  |  |  |  | Adduction | Flow |  |  |
|  |  |  |  | Abduction | Flow |  |  |
|  |  |  |  | Circumduction | Flow |  |  |
|  |  |  | Elbow | Extension | Flow | 12-30 |  |
|  |  |  |  | Flexion | Flow |  |  |
|  |  |  | Shoulder | Extension | Flow | 12-20 |  |
|  |  |  |  | Flexion | Flow |  |  |
|  |  |  |  | Adduction | Flow |  |  |
|  |  |  |  | Abduction | Flow |  |  |
|  |  |  |  | Horizontal adduction | Flow |  |  |
|  |  |  |  | Horizontal abduction | Flow |  |  |
|  |  |  |  | Shoulder girdle elevation/depression | Flow |  |  |
|  |  |  |  | Rotation (without arms) | Flow |  |  |
|  |  |  | Neck | Extension | Flow | 12-20 |  |
|  |  |  |  | Flexion | Flow |  |  |
|  |  |  |  | Lateral extension | Flow |  |  |
|  |  |  |  | Rotation | Flow |  |  |
|  |  |  | Torso | Fontal extension | Flow | 12-20 |  |
|  |  |  |  | Dorsal extension | Flow |  |  |
|  |  |  |  | Lateral extension | Flow |  |  |
|  |  |  |  | Rotation | Flow |  |  |
|  | **Lower Body** | 5 | Hip | Flexion | Flow | 12-20 |  |
|  |  |  |  | Extension | Flow |  |  |
|  |  |  |  | Adduction | Flow |  |  |
|  |  |  |  | Abduction | Flow |  |  |
|  |  |  |  | Internal rotation | Flow |  |  |
|  |  |  |  | External rotation | Flow |  |  |
|  |  |  | Knee | Extension | Flow | 12-20 |  |
|  |  |  |  | Flexion | Flow |  |  |
|  |  |  | Ankle | Extension | Flow | 12-30 |  |
|  |  |  |  | Flexion | Flow |  |  |
|  |  |  |  | Circumduction | Flow |  |  |
|  |  |  | Toes | Flexion | Flow | 12-30 |  |
|  |  |  |  | Extension | Flow |  |  |

| **M2M Week 3** | | **Duration**  **(minute)** | **Body Part** | **Muscle** | **Repetition** | **Set** | **Contraction/Muscle** | **Pattern** | **Tempo**  **(bpm)** | **Check** |
| --- | --- | --- | --- | --- | --- | --- | --- | --- | --- | --- |
| **Strength** | **Upper Body** | 5 | Shoulder | Deltoids | 8 | 1 | 8 | Linear | 40-60 |  |
|  |  |  | Chest | Trapezius/Rhomboids | 8 | 1 | 8 | Linear | 40-60 |  |
|  |  |  |  | Pectorals | 8 | 1 | 8 | Linear | 40-60 |  |
|  |  |  | Arm | Biceps | 8 | 1 | 8 | Linear | 40-80 |  |
|  |  |  |  | Triceps | 8 | 1 | 8 | Linear | 40-80 |  |
|  | **Lower Body** |  | Trunk | Erector Spinae | 8 | 1 | 8 | Linear | 40-60 |  |
|  |  |  |  | Rectus abdominus | 8 | 1 | 8 | Linear | 40-80 |  |
|  |  |  |  | Obliques | 8 | 1 | 8 | Linear | 40-80 |  |

| **M2M Week 3** | **Duration**  **(minute)** | **Movement** | **Pattern** | **Repetition** | **Set** | **Contraction/Movement** | **Tempo**  **(bpm)** | **Check** |
| --- | --- | --- | --- | --- | --- | --- | --- | --- |
| **Functional Strength**  **/Balance** | 5 | Tendu | 1^st^ parallel front, side and back | 4-8/direction | 1-2/feet position | 8/feet position | 30-60 |  |
|  |  | Pile | 1^st^ & 2^nd^ parallel | 8/feet position | 1/feet position | 8/feet position | 30-60 |  |
|  |  | Eleve | 1^st^ & 2^nd^ parallel | 8/feet position | 1/feet position | 8/feet position | 30-60 |  |
|  |  | Static Balance | One leg balance |  |  |  |  |  |

| **M2M Week 4** | | **Four 8-count breathing cycles at the beginning** | | | | | |
| --- | --- | --- | --- | --- | --- | --- | --- |
|  |  | **Duration**  **(minute)** | **Body Part** | **Movement** | **Pattern** | **Tempo**  **(bpm)** | **Check** |
| **Range of Motion** | **Upper Body** | 5 | Hand | Extension | Flow | 12-30 |  |
|  |  |  |  | Flexion | Flow |  |  |
|  |  |  | Wrist | Extension | Flow |  |  |
|  |  |  |  | Flexion | Flow |  |  |
|  |  |  |  | Adduction | Flow |  |  |
|  |  |  |  | Abduction | Flow |  |  |
|  |  |  |  | Circumduction | Flow |  |  |
|  |  |  | Elbow | Extension | Flow | 12-30 |  |
|  |  |  |  | Flexion | Flow |  |  |
|  |  |  | Shoulder | Extension | Flow | 12-20 |  |
|  |  |  |  | Flexion | Flow |  |  |
|  |  |  |  | Adduction | Flow |  |  |
|  |  |  |  | Abduction | Flow |  |  |
|  |  |  |  | Horizontal adduction | Flow |  |  |
|  |  |  |  | Horizontal abduction | Flow |  |  |
|  |  |  |  | Shoulder girdle elevation/depression | Flow |  |  |
|  |  |  |  | Rotation (without arms) | Flow |  |  |
|  |  |  | Neck | Extension | Flow | 12-20 |  |
|  |  |  |  | Flexion | Flow |  |  |
|  |  |  |  | Lateral extension | Flow |  |  |
|  |  |  |  | Rotation | Flow |  |  |
|  |  |  | Torso | Fontal extension | Flow | 12-20 |  |
|  |  |  |  | Dorsal extension | Flow |  |  |
|  |  |  |  | Lateral extension | Flow |  |  |
|  |  |  |  | Rotation | Flow |  |  |
|  | **Lower Body** | 5 | Hip | Flexion | Flow | 12-20 |  |
|  |  |  |  | Extension | Flow |  |  |
|  |  |  |  | Adduction | Flow |  |  |
|  |  |  |  | Abduction | Flow |  |  |
|  |  |  |  | Internal rotation | Flow |  |  |
|  |  |  |  | External rotation | Flow |  |  |
|  |  |  | Knee | Extension | Flow | 12-20 |  |
|  |  |  |  | Flexion | Flow |  |  |
|  |  |  | Ankle | Extension | Flow | 12-30 |  |
|  |  |  |  | Flexion | Flow |  |  |
|  |  |  |  | Circumduction | Flow |  |  |
|  |  |  | Toes | Flexion | Flow | 12-30 |  |
|  |  |  |  | Extension | Flow |  |  |

| **M2M Week 4** | | **Duration**  **(minute)** | **Body Part** | **Muscle** | **Repetition** | **Set** | **Contraction/Muscle** | **Pattern** | **Tempo**  **(bpm)** | **Check** |
| --- | --- | --- | --- | --- | --- | --- | --- | --- | --- | --- |
| **Strength** | **Upper Body** | 5 | Shoulder | Deltoids | 8 | 1 | 8 | Linear | 40-60 |  |
|  |  |  | Chest | Trapezius/Rhomboids | 8 | 1 | 8 | Linear | 40-60 |  |
|  |  |  |  | Pectorals | 8 | 1 | 8 | Linear | 40-60 |  |
|  |  |  | Arm | Biceps | 8 | 1 | 8 | Linear | 40-80 |  |
|  |  |  |  | Triceps | 8 | 1 | 8 | Linear | 40-80 |  |
|  | **Lower Body** |  | Trunk | Erector Spinae | 8 | 1 | 8 | Linear | 40-60 |  |
|  |  |  |  | Rectus abdominus | 8 | 1 | 8 | Linear | 40-80 |  |
|  |  |  |  | Obliques | 8 | 1 | 8 | Linear | 40-80 |  |

| **M2M Week 4** | **Time (minute)** | **Movement** | **Repetition** | **Set** | **Position** | **Pattern** | **Equipment** | **Tempo (movement)** | **Check** |
| --- | --- | --- | --- | --- | --- | --- | --- | --- | --- |
| **Cardio** | 5 | Any | - | - | Sitting | Constant move | Chair | 100-180 |  |

| **M2M Week 4** | **Duration**  **(minute)** | **Movement** | **Pattern** | **Repetition** | **Set** | **Contraction/Movement** | **Tempo**  **(bpm)** | **Check** |
| --- | --- | --- | --- | --- | --- | --- | --- | --- |
| **Functional Strength**  **/Balance** | 5 | Tendu | 1^st^ parallel front, side and back | 4-8/direction | 1-2/feet position | 8/feet position | 30-60 |  |
|  |  | Pile | 1^st^ & 2^nd^ parallel | 8/feet position | 1/feet position | 8/feet position | 30-60 |  |
|  |  | Eleve | 1^st^ & 2^nd^ parallel | 8/feet position | 1/feet position | 8/feet position | 30-60 |  |
|  |  | Static Balance | One leg balance |  |  |  |  |  |

| **M2M Week 4** | **Duration (minute)** | **Movement** | **Pattern** | **Repetition** | **Set** | **Position** | **Check** |
| --- | --- | --- | --- | --- | --- | --- | --- |
| **Cool Down** | 5 | Lateral breathing | Linear | - | - | Standing  /Sitting |  |
|  |  | Breathing with neck stretch | Linear | - | - | Standing  /Sitting |  |
|  |  | Breathing with torso stretch | Linear | - | - | Standing  /Sitting |  |
|  |  | Breathing with arm movement | Linear | - | - | Standing  /Sitting |  |

| **M2M Week 5** | | **Four 8-count breathing cycles at the beginning** | | | | | |
| --- | --- | --- | --- | --- | --- | --- | --- |
|  |  | **Duration**  **(minute)** | **Body Part** | **Movement** | **Pattern** | **Tempo**  **(bpm)** | **Check** |
| **Range of Motion** | **Upper Body** | 5 | Hand | Extension | Flow | 12-30 |  |
|  |  |  |  | Flexion | Flow |  |  |
|  |  |  | Wrist | Extension | Flow |  |  |
|  |  |  |  | Flexion | Flow |  |  |
|  |  |  |  | Adduction | Flow |  |  |
|  |  |  |  | Abduction | Flow |  |  |
|  |  |  |  | Circumduction | Flow |  |  |
|  |  |  | Elbow | Extension | Flow | 12-30 |  |
|  |  |  |  | Flexion | Flow |  |  |
|  |  |  | Shoulder | Extension | Flow | 12-20 |  |
|  |  |  |  | Flexion | Flow |  |  |
|  |  |  |  | Adduction | Flow |  |  |
|  |  |  |  | Abduction | Flow |  |  |
|  |  |  |  | Horizontal adduction | Flow |  |  |
|  |  |  |  | Horizontal abduction | Flow |  |  |
|  |  |  |  | Shoulder girdle elevation/depression | Flow |  |  |
|  |  |  |  | Rotation (without arms) | Flow |  |  |
|  |  |  | Neck | Extension | Flow | 12-20 |  |
|  |  |  |  | Flexion | Flow |  |  |
|  |  |  |  | Lateral extension | Flow |  |  |
|  |  |  |  | Rotation | Flow |  |  |
|  |  |  | Torso | Fontal extension | Flow | 12-20 |  |
|  |  |  |  | Dorsal extension | Flow |  |  |
|  |  |  |  | Lateral extension | Flow |  |  |
|  |  |  |  | Rotation | Flow |  |  |
|  | **Lower Body** | 5 | Hip | Flexion | Flow | 12-20 |  |
|  |  |  |  | Extension | Flow |  |  |
|  |  |  |  | Adduction | Flow |  |  |
|  |  |  |  | Abduction | Flow |  |  |
|  |  |  |  | Internal rotation | Flow |  |  |
|  |  |  |  | External rotation | Flow |  |  |
|  |  |  | Knee | Extension | Flow | 12-20 |  |
|  |  |  |  | Flexion | Flow |  |  |
|  |  |  | Ankle | Extension | Flow | 12-30 |  |
|  |  |  |  | Flexion | Flow |  |  |
|  |  |  |  | Circumduction | Flow |  |  |
|  |  |  | Toes | Flexion | Flow | 12-30 |  |
|  |  |  |  | Extension | Flow |  |  |

| **M2M Week 5** | | **Duration**  **(minute)** | **Body Part** | **Muscle** | **Repetition** | **Set** | **Contraction/Muscle** | **Pattern** | **Tempo**  **(bpm)** | **Check** |
| --- | --- | --- | --- | --- | --- | --- | --- | --- | --- | --- |
| **Strength** | **Upper Body** | 5 | Shoulder | Deltoids | 5 | 2 | 10 | Small Variation | 40-60 |  |
|  |  |  | Chest | Trapezius/Rhomboids | 5 | 2 | 10 | Small Variation | 40-60 |  |
|  |  |  |  | Pectorals | 5 | 2 | 10 | Small Variation | 40-60 |  |
|  |  |  | Arm | Biceps | 5 | 2 | 10 | Small Variation | 40-80 |  |
|  |  |  |  | Triceps | 5 | 2 | 10 | Small Variation | 40-80 |  |
|  | **Lower Body** |  | Trunk | Erector Spinae | 5 | 2 | 10 | Small Variation | 40-60 |  |
|  |  |  |  | Rectus abdominus | 5 | 2 | 10 | Small Variation | 40-80 |  |
|  |  |  |  | Obliques | 5 | 2 | 10 | Small Variation | 40-80 |  |

| **M2M Week 5** | **Time (minute)** | **Movement** | **Repetition** | **Set** | **Position** | **Pattern** | **Equipment** | **Tempo (movement)** | **Check** |
| --- | --- | --- | --- | --- | --- | --- | --- | --- | --- |
| **Cardio** | 5 | Any | - | - | Sitting | Constant move | Chair | 100-180 |  |

| **M2M Week 5** | **Duration**  **(minute)** | **Movement** | **Pattern** | **Repetition** | **Set** | **Contraction/Movement** | **Tempo**  **(bpm)** | **Check** |
| --- | --- | --- | --- | --- | --- | --- | --- | --- |
| **Functional Strength**  **/Balance** | 5 | Pile | 1^st^ & 2^nd^ parallel | 8/feet position | 1/feet position | 8/feet position | 30-60 |  |
|  |  | Eleve | 1^st^ & 2^nd^ parallel | 8/feet position | 1/feet position | 8/feet position | 30-60 |  |
|  |  | Weight Shifting | 3 directions (front, side, back and diagonal) both legs; keep supporting leg on the ground |  |  |  |  |  |
|  |  | Static Balance | One leg balance |  |  |  |  |  |

| **M2M Week 5** | **Duration (minute)** | **Movement** | **Pattern** | **Repetition** | **Set** | **Position** | **Check** |
| --- | --- | --- | --- | --- | --- | --- | --- |
| **Cool Down** | 5 | Lateral breathing | Linear | - | - | Standing  /Sitting |  |
|  |  | Breathing with neck stretch | Linear | - | - | Standing  /Sitting |  |
|  |  | Breathing with torso stretch | Linear | - | - | Standing  /Sitting |  |
|  |  | Breathing with arm movement | Linear | - | - | Standing  /Sitting |  |

| **M2M Week 6** | | **Four 8-count breathing cycles at the beginning** | | | | | |
| --- | --- | --- | --- | --- | --- | --- | --- |
|  |  | **Duration**  **(minute)** | **Body Part** | **Movement** | **Pattern** | **Tempo**  **(bpm)** | **Check** |
| **Range of Motion** | **Upper Body** | 5 | Hand | Extension | Flow | 12-30 |  |
|  |  |  |  | Flexion | Flow |  |  |
|  |  |  | Wrist | Extension | Flow |  |  |
|  |  |  |  | Flexion | Flow |  |  |
|  |  |  |  | Adduction | Flow |  |  |
|  |  |  |  | Abduction | Flow |  |  |
|  |  |  |  | Circumduction | Flow |  |  |
|  |  |  | Elbow | Extension | Flow | 12-30 |  |
|  |  |  |  | Flexion | Flow |  |  |
|  |  |  | Shoulder | Extension | Flow | 12-20 |  |
|  |  |  |  | Flexion | Flow |  |  |
|  |  |  |  | Adduction | Flow |  |  |
|  |  |  |  | Abduction | Flow |  |  |
|  |  |  |  | Horizontal adduction | Flow |  |  |
|  |  |  |  | Horizontal abduction | Flow |  |  |
|  |  |  |  | Shoulder girdle elevation/depression | Flow |  |  |
|  |  |  |  | Rotation (without arms) | Flow |  |  |
|  |  |  | Neck | Extension | Flow | 12-20 |  |
|  |  |  |  | Flexion | Flow |  |  |
|  |  |  |  | Lateral extension | Flow |  |  |
|  |  |  |  | Rotation | Flow |  |  |
|  |  |  | Torso | Fontal extension | Flow | 12-20 |  |
|  |  |  |  | Dorsal extension | Flow |  |  |
|  |  |  |  | Lateral extension | Flow |  |  |
|  |  |  |  | Rotation | Flow |  |  |
|  | **Lower Body** | 5 | Hip | Flexion | Flow | 12-20 |  |
|  |  |  |  | Extension | Flow |  |  |
|  |  |  |  | Adduction | Flow |  |  |
|  |  |  |  | Abduction | Flow |  |  |
|  |  |  |  | Internal rotation | Flow |  |  |
|  |  |  |  | External rotation | Flow |  |  |
|  |  |  | Knee | Extension | Flow | 12-20 |  |
|  |  |  |  | Flexion | Flow |  |  |
|  |  |  | Ankle | Extension | Flow | 12-30 |  |
|  |  |  |  | Flexion | Flow |  |  |
|  |  |  |  | Circumduction | Flow |  |  |
|  |  |  | Toes | Flexion | Flow | 12-30 |  |
|  |  |  |  | Extension | Flow |  |  |

| **M2M Week 6** | | **Duration**  **(minute)** | **Body Part** | **Muscle** | **Repetition** | **Set** | **Contraction/Muscle** | **Pattern** | **Tempo**  **(bpm)** | **Check** |
| --- | --- | --- | --- | --- | --- | --- | --- | --- | --- | --- |
| **Strength** | **Upper Body** | 5 | Shoulder | Deltoids | 4 | 3 | 12 | Small Variation | 40-60 |  |
|  |  |  | Chest | Trapezius/Rhomboids | 4 | 3 | 12 | Small Variation | 40-60 |  |
|  |  |  |  | Pectorals | 4 | 3 | 12 | Small Variation | 40-60 |  |
|  |  |  | Arm | Biceps | 4 | 3 | 12 | Small Variation | 40-80 |  |
|  |  |  |  | Triceps | 4 | 3 | 12 | Small Variation | 40-80 |  |
|  | **Lower Body** |  | Trunk | Erector Spinae | 4 | 3 | 12 | Small Variation | 40-60 |  |
|  |  |  |  | Rectus abdominus | 4 | 3 | 12 | Small Variation | 40-80 |  |
|  |  |  |  | Obliques | 4 | 3 | 12 | Small Variation | 40-80 |  |

| **M2M Week 6** | **Time (minute)** | **Movement** | **Repetition** | **Set** | **Position** | **Pattern** | **Equipment** | **Tempo (movement)** | **Check** |
| --- | --- | --- | --- | --- | --- | --- | --- | --- | --- |
| **Cardio** | 15 | Any | - | - | Sitting | Constant move | Chair | 100-180 |  |

| **M2M Week 6** | **Duration**  **(minute)** | **Movement** | **Pattern** | **Repetition** | **Set** | **Contraction/Movement** | **Tempo**  **(bpm)** | **Check** |
| --- | --- | --- | --- | --- | --- | --- | --- | --- |
| **Functional Strength**  **/Balance** | 5 | Pile | 1^st^ & 2^nd^ parallel | 8/feet position | 1/feet position | 8/feet position | 30-60 |  |
|  |  | Eleve | 1^st^ & 2^nd^ parallel | 8/feet position | 1/feet position | 8/feet position | 30-60 |  |
|  |  | Weight Shifting | 3 directions (front, side, back and diagonal) both legs; keep supporting leg on the ground |  |  |  |  |  |
|  |  | Static Balance | One leg balance |  |  |  |  |  |

| **M2M Week 6** | **Duration (minute)** | **Movement** | **Pattern** | **Repetition** | **Set** | **Position** | **Check** |
| --- | --- | --- | --- | --- | --- | --- | --- |
| **Cool Down** | 5 | Lateral breathing | Linear | - | - | Standing  /Sitting |  |
|  |  | Breathing with neck stretch | Linear | - | - | Standing  /Sitting |  |
|  |  | Breathing with torso stretch | Linear | - | - | Standing  /Sitting |  |
|  |  | Breathing with arm movement | Linear | - | - | Standing  /Sitting |  |

| **M2M Week 7** | | **Four 8-count breathing cycles at the beginning** | | | | | |
| --- | --- | --- | --- | --- | --- | --- | --- |
|  |  | **Duration**  **(minute)** | **Body Part** | **Movement** | **Pattern** | **Tempo**  **(bpm)** | **Check** |
| **Range of Motion** | **Upper Body** | 5 | Hand | Extension | Flow | 12-30 |  |
|  |  |  |  | Flexion | Flow |  |  |
|  |  |  | Wrist | Extension | Flow |  |  |
|  |  |  |  | Flexion | Flow |  |  |
|  |  |  |  | Adduction | Flow |  |  |
|  |  |  |  | Abduction | Flow |  |  |
|  |  |  |  | Circumduction | Flow |  |  |
|  |  |  | Elbow | Extension | Flow | 12-30 |  |
|  |  |  |  | Flexion | Flow |  |  |
|  |  |  | Shoulder | Extension | Flow | 12-20 |  |
|  |  |  |  | Flexion | Flow |  |  |
|  |  |  |  | Adduction | Flow |  |  |
|  |  |  |  | Abduction | Flow |  |  |
|  |  |  |  | Horizontal adduction | Flow |  |  |
|  |  |  |  | Horizontal abduction | Flow |  |  |
|  |  |  |  | Shoulder girdle elevation/depression | Flow |  |  |
|  |  |  |  | Rotation (without arms) | Flow |  |  |
|  |  |  | Neck | Extension | Flow | 12-20 |  |
|  |  |  |  | Flexion | Flow |  |  |
|  |  |  |  | Lateral extension | Flow |  |  |
|  |  |  |  | Rotation | Flow |  |  |
|  |  |  | Torso | Fontal extension | Flow | 12-20 |  |
|  |  |  |  | Dorsal extension | Flow |  |  |
|  |  |  |  | Lateral extension | Flow |  |  |
|  |  |  |  | Rotation | Flow |  |  |
|  | **Lower Body** | 5 | Hip | Flexion | Flow | 12-20 |  |
|  |  |  |  | Extension | Flow |  |  |
|  |  |  |  | Adduction | Flow |  |  |
|  |  |  |  | Abduction | Flow |  |  |
|  |  |  |  | Internal rotation | Flow |  |  |
|  |  |  |  | External rotation | Flow |  |  |
|  |  |  | Knee | Extension | Flow | 12-20 |  |
|  |  |  |  | Flexion | Flow |  |  |
|  |  |  | Ankle | Extension | Flow | 12-30 |  |
|  |  |  |  | Flexion | Flow |  |  |
|  |  |  |  | Circumduction | Flow |  |  |
|  |  |  | Toes | Flexion | Flow | 12-30 |  |
|  |  |  |  | Extension | Flow |  |  |

| **M2M Week 7** | | **Duration**  **(minute)** | **Body Part** | **Muscle** | **Repetition** | **Set** | **Contraction/Muscle** | **Pattern** | **Tempo**  **(bpm)** | **Check** |
| --- | --- | --- | --- | --- | --- | --- | --- | --- | --- | --- |
| **Strength** | **Upper Body** | 5 | Shoulder | Deltoids | 4 | 3 | 12 | Small Variation | 40-60 |  |
|  |  |  | Chest | Trapezius/Rhomboids | 4 | 3 | 12 | Small Variation | 40-60 |  |
|  |  |  |  | Pectorals | 4 | 3 | 12 | Small Variation | 40-60 |  |
|  |  |  | Arm | Biceps | 4 | 3 | 12 | Small Variation | 40-80 |  |
|  |  |  |  | Triceps | 4 | 3 | 12 | Small Variation | 40-80 |  |
|  | **Lower Body** |  | Trunk | Erector Spinae | 4 | 3 | 12 | Small Variation | 40-60 |  |
|  |  |  |  | Rectus abdominus | 4 | 3 | 12 | Small Variation | 40-80 |  |
|  |  |  |  | Obliques | 4 | 3 | 12 | Small Variation | 40-80 |  |

| **M2M Week 7** | **Time (minute)** | **Movement** | **Repetition** | **Set** | **Position** | **Pattern** | **Equipment** | **Tempo (movement)** | **Check** |
| --- | --- | --- | --- | --- | --- | --- | --- | --- | --- |
| **Cardio** | 15 | Any | - | - | Sitting | Constant move | Chair | 100-180 |  |

| **M2M Week 7** | **Duration**  **(minute)** | **Movement** | **Pattern** | **Repetition** | **Set** | **Contraction/Movement** | **Tempo**  **(bpm)** | **Check** |
| --- | --- | --- | --- | --- | --- | --- | --- | --- |
| **Functional Strength**  **/Balance** | 5 | Pile | 1^st^ & 2^nd^ parallel | 4/feet position | 1/feet position | 4/feet position | 30-60 |  |
|  |  | Eleve | 1^st^ & 2^nd^ parallel | 4/feet position | 1/feet position | 4/feet position | 30-60 |  |
|  |  | Degage | 1^st^ parallel front, side and back | 4/feet position | 1/feet position | 4/feet position | 30-60 |  |
|  |  | Weight Shifting | 3 directions (front, side, back and diagonal) both legs; bring supporting leg off ground |  |  |  |  |  |

| **M2M Week 7** | **Duration (minute)** | **Movement** | **Pattern** | **Repetition** | **Set** | **Position** | **Check** |
| --- | --- | --- | --- | --- | --- | --- | --- |
| **Cool Down** | 5 | Lateral breathing | Linear | - | - | Standing  /Sitting |  |
|  |  | Breathing with neck stretch | Linear | - | - | Standing  /Sitting |  |
|  |  | Breathing with torso stretch | Linear | - | - | Standing  /Sitting |  |
|  |  | Breathing with arm movement | Linear | - | - | Standing  /Sitting |  |

| **M2M Week 8** | | **Four 8-count breathing cycles at the beginning** | | | | | |
| --- | --- | --- | --- | --- | --- | --- | --- |
|  |  | **Duration**  **(minute)** | **Body Part** | **Movement** | **Pattern** | **Tempo**  **(bpm)** | **Check** |
| **Range of Motion** | **Upper Body** | 5 | Hand | Extension | Flow | 12-30 |  |
|  |  |  |  | Flexion | Flow |  |  |
|  |  |  | Wrist | Extension | Flow |  |  |
|  |  |  |  | Flexion | Flow |  |  |
|  |  |  |  | Adduction | Flow |  |  |
|  |  |  |  | Abduction | Flow |  |  |
|  |  |  |  | Circumduction | Flow |  |  |
|  |  |  | Elbow | Extension | Flow | 12-30 |  |
|  |  |  |  | Flexion | Flow |  |  |
|  |  |  | Shoulder | Extension | Flow | 12-20 |  |
|  |  |  |  | Flexion | Flow |  |  |
|  |  |  |  | Adduction | Flow |  |  |
|  |  |  |  | Abduction | Flow |  |  |
|  |  |  |  | Horizontal adduction | Flow |  |  |
|  |  |  |  | Horizontal abduction | Flow |  |  |
|  |  |  |  | Shoulder girdle elevation/depression | Flow |  |  |
|  |  |  |  | Rotation (without arms) | Flow |  |  |
|  |  |  | Neck | Extension | Flow | 12-20 |  |
|  |  |  |  | Flexion | Flow |  |  |
|  |  |  |  | Lateral extension | Flow |  |  |
|  |  |  |  | Rotation | Flow |  |  |
|  |  |  | Torso | Fontal extension | Flow | 12-20 |  |
|  |  |  |  | Dorsal extension | Flow |  |  |
|  |  |  |  | Lateral extension | Flow |  |  |
|  |  |  |  | Rotation | Flow |  |  |
|  | **Lower Body** | 5 | Hip | Flexion | Flow | 12-20 |  |
|  |  |  |  | Extension | Flow |  |  |
|  |  |  |  | Adduction | Flow |  |  |
|  |  |  |  | Abduction | Flow |  |  |
|  |  |  |  | Internal rotation | Flow |  |  |
|  |  |  |  | External rotation | Flow |  |  |
|  |  |  | Knee | Extension | Flow | 12-20 |  |
|  |  |  |  | Flexion | Flow |  |  |
|  |  |  | Ankle | Extension | Flow | 12-30 |  |
|  |  |  |  | Flexion | Flow |  |  |
|  |  |  |  | Circumduction | Flow |  |  |
|  |  |  | Toes | Flexion | Flow | 12-30 |  |
|  |  |  |  | Extension | Flow |  |  |

| **M2M Week 8** | | **Duration**  **(minute)** | **Body Part** | **Muscle** | **Repetition** | **Set** | **Contraction/Muscle** | **Pattern** | **Tempo**  **(bpm)** | **Check** |
| --- | --- | --- | --- | --- | --- | --- | --- | --- | --- | --- |
| **Strength** | **Upper Body** | 5 | Shoulder | Deltoids | 4 | 3 | 12 | Small Variation | 40-60 |  |
|  |  |  | Chest | Trapezius/Rhomboids | 4 | 3 | 12 | Small Variation | 40-60 |  |
|  |  |  |  | Pectorals | 4 | 3 | 12 | Small Variation | 40-60 |  |
|  |  |  | Arm | Biceps | 4 | 3 | 12 | Small Variation | 40-80 |  |
|  |  |  |  | Triceps | 4 | 3 | 12 | Small Variation | 40-80 |  |
|  | **Lower Body** |  | Trunk | Erector Spinae | 4 | 3 | 12 | Small Variation | 40-60 |  |
|  |  |  |  | Rectus abdominus | 4 | 3 | 12 | Small Variation | 40-80 |  |
|  |  |  |  | Obliques | 4 | 3 | 12 | Small Variation | 40-80 |  |

| **M2M Week 8** | **Time (minute)** | **Movement** | **Repetition** | **Set** | **Position** | **Pattern** | **Equipment** | **Tempo (movement)** | **Check** |
| --- | --- | --- | --- | --- | --- | --- | --- | --- | --- |
| **Cardio** | 15 | Any | - | - | Sitting | Constant move | Chair | 100-180 |  |

| **M2M Week 8** | **Duration**  **(minute)** | **Movement** | **Pattern** | **Repetition** | **Set** | **Contraction/Movement** | **Tempo**  **(bpm)** | **Check** |
| --- | --- | --- | --- | --- | --- | --- | --- | --- |
| **Functional Strength**  **/Balance** | 5 | Pile | 1^st^ & 2^nd^ parallel | 4/feet position | 1/feet position | 4/feet position | 30-60 |  |
|  |  | Eleve | 1^st^ & 2^nd^ parallel | 4/feet position | 1/feet position | 4/feet position | 30-60 |  |
|  |  | Degage | 1^st^ parallel front, side and back | 4/feet position | 1/feet position | 4/feet position | 30-60 |  |
|  |  | Weight Shifting | 3 directions (front, side, back and diagonal) both legs; bring supporting leg off ground |  |  |  |  |  |

| **M2M Week 8** | **Duration (minute)** | **Movement** | **Pattern** | **Repetition** | **Set** | **Position** | **Check** |
| --- | --- | --- | --- | --- | --- | --- | --- |
| **Cool Down** | 5 | Lateral breathing | Linear | - | - | Standing  /Sitting |  |
|  |  | Breathing with neck stretch | Linear | - | - | Standing  /Sitting |  |
|  |  | Breathing with torso stretch | Linear | - | - | Standing  /Sitting |  |
|  |  | Breathing with arm movement | Linear | - | - | Standing  /Sitting |  |
